# DFT-based prediction of reactivity of short-chain alcohol dehydrogenase

**DOI:** 10.1007/s10822-017-0026-5

**Published:** 2017-05-26

**Authors:** I. Stawoska, A. Dudzik, M. Wasylewski, M. Jemioła-Rzemińska, A. Skoczowski, K. Strzałka, M. Szaleniec

**Affiliations:** 1 Institute of Biology, Pedagogical University of Cracow, Podchorążych 2, 30-084 Kraków, Poland; 2 Jerzy Haber Institute of Catalysis and Surface Chemistry, Polish Academy of Sciences, Niezapominajek 8, 30-239 Kraków, Poland; 3 Faculty of Biochemistry, Biophysics and Biotechnology, Jagiellonian University, Gronostajowa 7, 30-387 Kraków, Poland; 4 Malopolska Centre of Biotechnology, Jagiellonian University, Gronostajowa 7A, 30-387 Kraków, Poland; 5 The Franciszek Górski Institute of Plant Physiology, Polish Academy of Sciences, Niezapominajek 21, 30-239 Kraków, Poland

**Keywords:** Short chain dehydrogenase, (*S*)-1-phenylethanol dehydrogenase, PEDH, Alcohol dehydrogenase/ketoreductase, Reduction of ketones, Hydride transfer

## Abstract

The reaction mechanism of ketone reduction by short chain dehydrogenase/reductase, (*S*)-1-phenylethanol dehydrogenase from *Aromatoleum aromaticum*, was studied with DFT methods using cluster model approach. The characteristics of the hydride transfer process were investigated based on reaction of acetophenone and its eight structural analogues. The results confirmed previously suggested concomitant transfer of hydride from NADH to carbonyl C atom of the substrate with proton transfer from Tyr to carbonyl O atom. However, additional coupled motion of the next proton in the proton-relay system, between O2′ ribose hydroxyl and Tyr154 was observed. The protonation of Lys158 seems not to affect the pKa of Tyr154, as the stable tyrosyl anion was observed only for a neutral Lys158 in the high pH model. The calculated reaction energies and reaction barriers were calibrated by calorimetric and kinetic methods. This allowed an excellent prediction of the reaction enthalpies (R^2^ = 0.93) and a good prediction of the reaction kinetics (R^2^ = 0.89). The observed relations were validated in prediction of log *K*_*eq*_ obtained for real whole-cell reactor systems that modelled industrial synthesis of *S*-alcohols.

**Electronic supplementary material:**

The online version of this article (doi:10.1007/s10822-017-0026-5) contains supplementary material, which is available to authorized users.

## Introduction

**Fig. 1:**
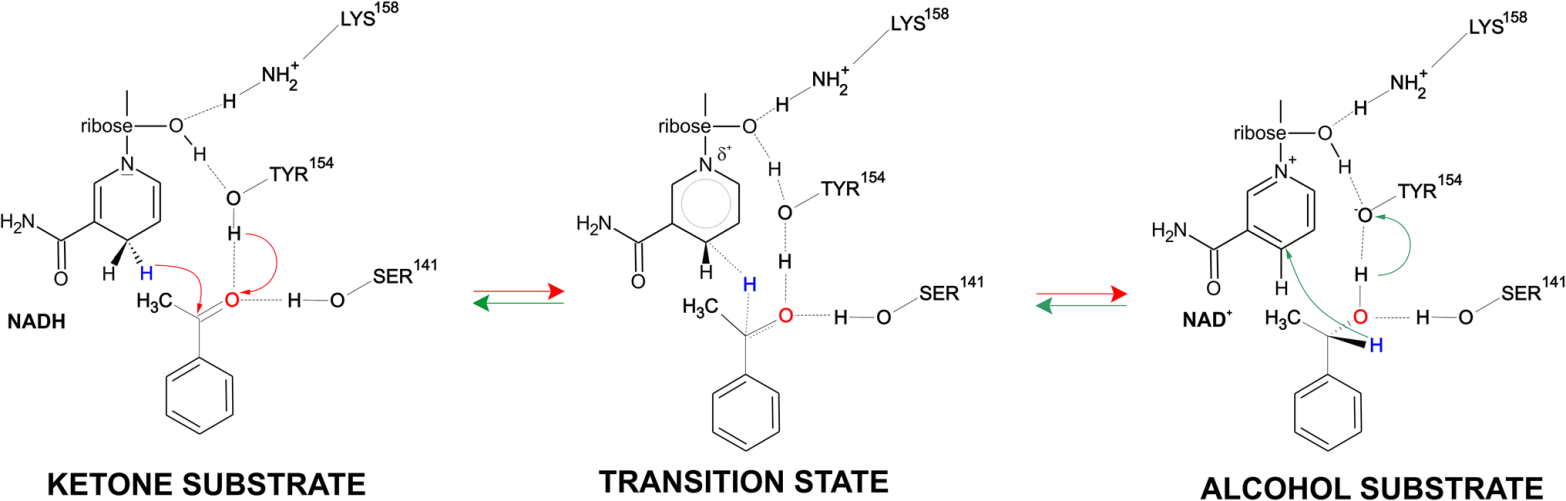
Reaction mechanism postulated for short-chain alcohol dehydrogenase

Pure enantiomers of chiral alcohols are one of the most valuable synthons for the production of various biologically active compounds especially pharmaceuticals, agrochemicals or flavours [1–6]. A straightforward approach to the synthesis of chiral alcohols is the asymmetric reduction of corresponding carbonyl compounds, which can be achieved by chemical or biocatalytic methods. Biocatalytic methods offer potentially lower cost and higher enantioselectivity of the process comparing with traditional chemical synthesis. One of the successful examples of commercialized biocatalytic methods for synthesis of chiral alcohols is (*S*)-1-phenylethanol dehydrogenase (PEDH) from *Aromatoleum aromaticum* (strain EbN1) [7–12]. It catalyses the NAD^+^ dependent stereospecific oxidation of (*S*)-1-phenylethanol to acetophenone during anaerobic ethylbenzene mineralization as well as the reverse reaction: the NADH dependent enantioselective reduction of acetophenone to (*S*)-1-phenylethanol (Fig. 1). The latter reaction is particularly interesting since such alcohols are important substances for pharmaceutical, fragrance or food industry [13]. The reaction mechanism postulated for short-chain alcohol dehydrogenases assumes ordered ternary complex kinetic mechanism i.e., the order binding of NAD^+^/NADH to the active site followed by binding of the substrate [14]. The enantioselectivity of the reaction is enforced by recognition of the bigger and smaller substituents (in this case phenyl and methyl group) as well as H-bonding interactions of the keto/hydroxy group with Tyr and Ser residues. It is postulated that at high pH the Tyr residue is present as a tyrosyl anion (TyrO^−^), which promotes proton abstraction from the hydroxyl group during hydride shift from the oxidized carbon atom to NAD^+^. The tyrosyl anion form is putatively stabilized by positive charges of nicotinamide ring of NAD^+^ as well as NεH_3_^+^ group of Lys, that is involved in proton relay system [15]. On the other hand in the acidic pH the Tyr residue is predominantly neutral, which again promotes protonation of the ketone group during hydride shift from NADH to carbonyl atom of the reagent [8].

PEDH is a short-chain dehydrogenase/reductase with a homotetrameric structure which was characterized by means of protein crystallography [7]. This finding allows to apply quantum chemical methods to describe the enzyme structure and function. Among important scientific and practical challenges, in terms of application of alcohol dehydrogenases in organic synthesis, is an efficient prediction of the reaction rates and equilibria with respect to the structure of the reactant. The previously developed statistical artificial neural network models required huge number of experimental data in order to approximate PEDH performance in reduction of new ketones in reactor conditions [8]. The other way to address this issue is to use cluster models that can account for pH shift of the system (i.e., include the H-bond transfer chain present in alcohol dehydrogenases and pH sensitive residues) and quantum chemical methods for prediction of both energy barriers of the hydride transfer and overall energetics of the ketone reduction process. However, as cluster models are far too simple to deliver an exact values of transition state barriers or reaction thermodynamics the experimental verification and calibration of the obtained results is required. To reach this goal kinetic and thermodynamic experiments were conducted for a series of nine substrates which differed in reaction rates and observed equilibria of ketone reduction. Both spectroscopic kinetic techniques and isothermal titration calorimetric method were used to obtain experimental parameters and the results of these experiments were correlated with results of molecular modelling. Finally, the obtained relations were tested in prediction of equilibria in the real whole-cell reactor systems, which modelled industrial reactor systems.

## Materials and methods

### Reagents

All reagents were obtained from Sigma–Aldrich, Fluka (Germany). The recombinant PEDH from *Aromatoleum aromaticum* was overexpressed in *Escherichia coli* [16] and isolated according to the previously described protocol [8]. The solution of the enzyme (app. 2 mg ml^−1^) was obtained in water and its concentration was determined with Bradford assay. 10% of glycerol was added to the protein samples before freezing in −20 °C.

### Enzyme assay—kinetic measurements

PEDH for enzymatic assay was purified according to the procedure described previously [8]. Ketone reduction activity of PEDH was initiated by addition of the selected substrate (acetophenone, 2,2-di-chloroacetophenone, 2-chloroacetophenone, 4′-nitroacetophenone, 4′-hydroxyacetophenone, 4′-chloroacetophenone, 4′-bromoacetophenone as well as 2- and 4-acetylpyridine) from stock solution in acetonitrile (the final concentration was 0.5 mM), and NADH oxidation was followed at 365 nm (Δε = 3.4 × 10^−3^ M^−1^ cm^−1^).

The experiments were performed at an optimum pH of 5.5 at 30 °C in 0.5 ml quartz cuvettes, in 100 mM MES/KOH buffer containing 0.5 mM NADH and 5–10 μl of purified PEDH (app. 2 mg ml^−1^). To determine changes in absorbance Lambda Bio 25 (Perkin-Elmer) spectrophotometer was used.

### Isothermal titration calorimetry, ITC

The tested reactions catalysed by PEDH were monitored by measuring the heat released upon the reaction progress using an ITC NANO 2G calorimeter (TA Instruments, USA). The apparatus had a 950 µl sample cell and a 50 µl dosing syringe, the measurement temperature was 25 °C. During all the experiments, the reference cell was filled with deionized water. Before starting the measurement, the apparatus was equilibrated at a desired temperature. The stirring rate provided by the injector paddle rotation was set at 250 rpm. In the performed experiments, the reaction cell was filled with PEDH (titrand, 2 × 10^−3^ mg ml^−1^) solution in 100 mM MES buffer pH 5.5, containing NADH (0.5 mM) and 10% (v/v) of acetonitrile. The titrant (the substrate) was dissolved in acetonitrile (HPLC grade) and mixed with the MES buffer (100 mM, pH 5.5, 1:9 v/v) to the final concentration of 125 µM. All the solutions were degassed at ca 40 kPa for 10–15 min using Degassing Station (TA Instruments). The combined reaction and mixing heats were established by a single injection of the tested substrate, i.e., acetophenone and its eight different structural analogues (Fig. 3) into the PEDH solution. The standard volume of the substrate solution introduced to the reaction cell was 20 µl with the exception of 4′-hydroxyacetophenone for which a 15 µl injection was used in order to decrease the experiment time to approx. 4000 s. The reaction progress was monitored by heat flow. In order to eliminate the heat effects which were not responsible for the reaction progress (i.e., the heat of reactant mixing, the heat of substrate solvatation), reference experiments were performed (injection of the tested substrate to the solution without PEDH). The average values obtained for blank experiments were subtracted from the values of PEDH experiments. All the tests were performed in 2–4 replicates.

The ITC signal processing (i.e., baseline subtraction, peak integration) was performed using the NanoAnalyze™ Software (TA Instruments). To calculate the heat effect of each process, the linear baseline was subtracted from each curve to account for the instrument thermal drift. Immediately after the experiments, samples were removed from the calorimeter and stored at −80 °C prior to a quantitative HPLC analysis.

### HPLC

In order to calculate the reactant and the product concentrations, the samples were analysed with DAD-HPLC (Agilent 1100) as described previously [8].

### Preparation of the cluster model

**Fig. 2:**
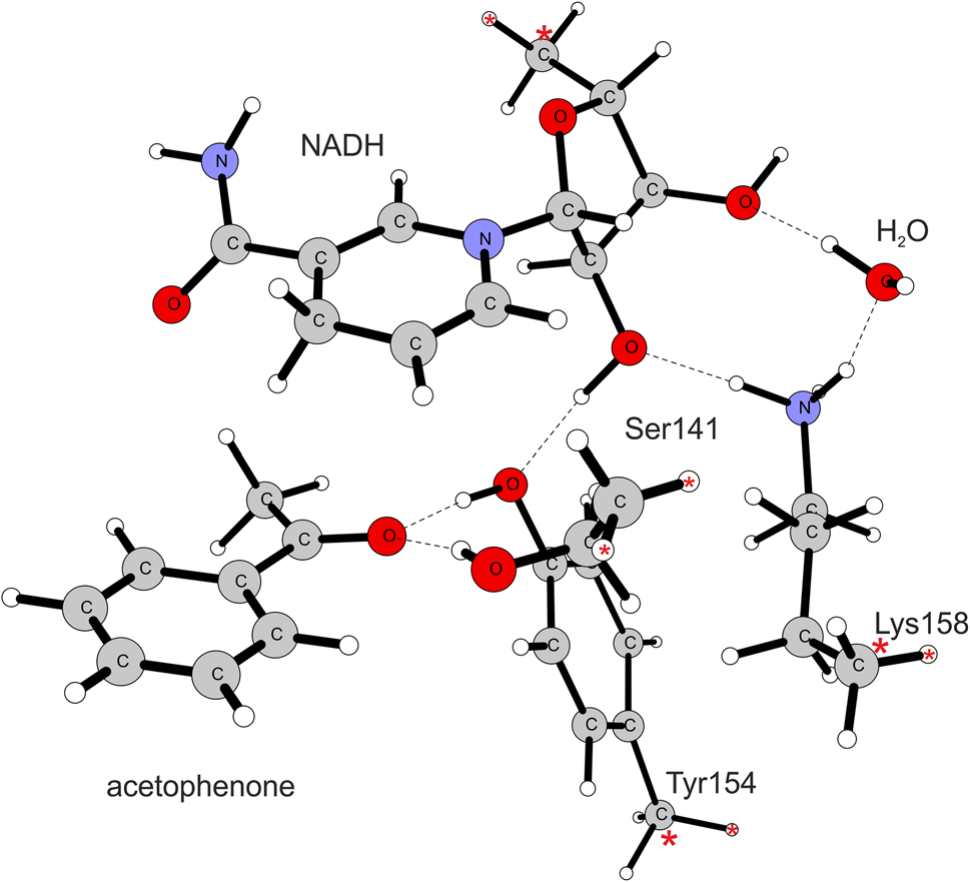
The low pH cluster model (protonated Lys158). The *dashed lines* indicate hydrogen bonds while *red asterisks mark* atoms with frozen coordinates

The quantum chemical modelling of the PEDH reaction mechanism was conducted within DFT theory using cluster model approach. The model was developed on the basis of the crystal structure of PEDH deposited in the PDB database (PDB code 2EWM). The acetophenone was docked into the active site using previously described protocol [8] and the initial geometry of the enzyme-substrate complex was minimized with CHARMm force field in Discovery Studio 4.0 (Biovia) [17] with 0.004 kJ mol^−1^ gradient tolerance using a distance-dependent dielectric model solvent (ε = 4). The cluster model accounted for those amino acids that are involved in H-bond interaction with a substrate and are essential to the catalytic process as well as the residues involved in proton transfer chain to the solvent. As a result, the model consisted of the substrate (acetophenone or its analogue), a fragment of the NADH cofactor (a ring of nicotinamide acid and ribose ring), residues of amino acids of the catalytic triad (Tyr154, Ser141, Lys158) and a water molecule that formed a hydrogen-bond with γ-NH_3_-Lys group. The Lys158 was in either protonated or deprotonated form, thus representing in turn low or high pH. Such protonation aimed at modelling the reaction optimal conditions for reduction of ketones (pH 5.5) or oxidation of alcohols (pH 8.5), respectively. This is due to the fact that the higher concentration of protons favours the reduction process which consumes one proton, whereas a deprotonated active site promotes the oxidation reaction which produces a proton. The truncated bonds were caped with hydrogen atoms and their coordinates as well as the coordinates of the adjacent carbon atoms were frozen as indicated on Fig. 2. The only exception was a residue of Ser141 where H atoms were introduced in place of the backbone C and N atoms and their coordinates were frozen.

### Computational procedures

The DFT calculations were conducted with Gaussian 09 programme [18]. For the initial model evaluation (i.e., reactions with acetophenone with high and low pH models) the restricted B3LYP [19] and restricted X3LYP [20] functionals were used. The geometry optimization was conducted in vacuum with 6–31(*d,p*) basis set and transition states were localized in relaxed potential energy scans along the reaction coordinate (i.e., NAD-H–C=O distance) followed by optimization of TS with Berny algorithm. The electronic energy of stationary points was corrected with single point calculation on 6-311 +g(*2d,2p*) theory while corrections for the effect of the solvent were obtained with single point calculation with PCM model solvent (ε = 4.0) on 6-31(*d,p*) level of theory according to previously validated protocols [21]. The vibration analysis conducted at B3LYP(X3LYP)/6-31g(*d,p*) level was used to obtain ZPE, enthalpy and Gibbs free energy corrections for each of the stationary points. The vibrational corrections were calculated for standard conditions (1 atm., 298 K, no scaling factor) with constrains introduced in Cartesian coordinates.

Finally, the vdW dispersion effects were evaluated for B3LYP functional for stationary point using Grimme D approach [22] implemented in XYZ-Viewer (s6 = 1.05, damping factor α = 20).

**Fig. 3:**
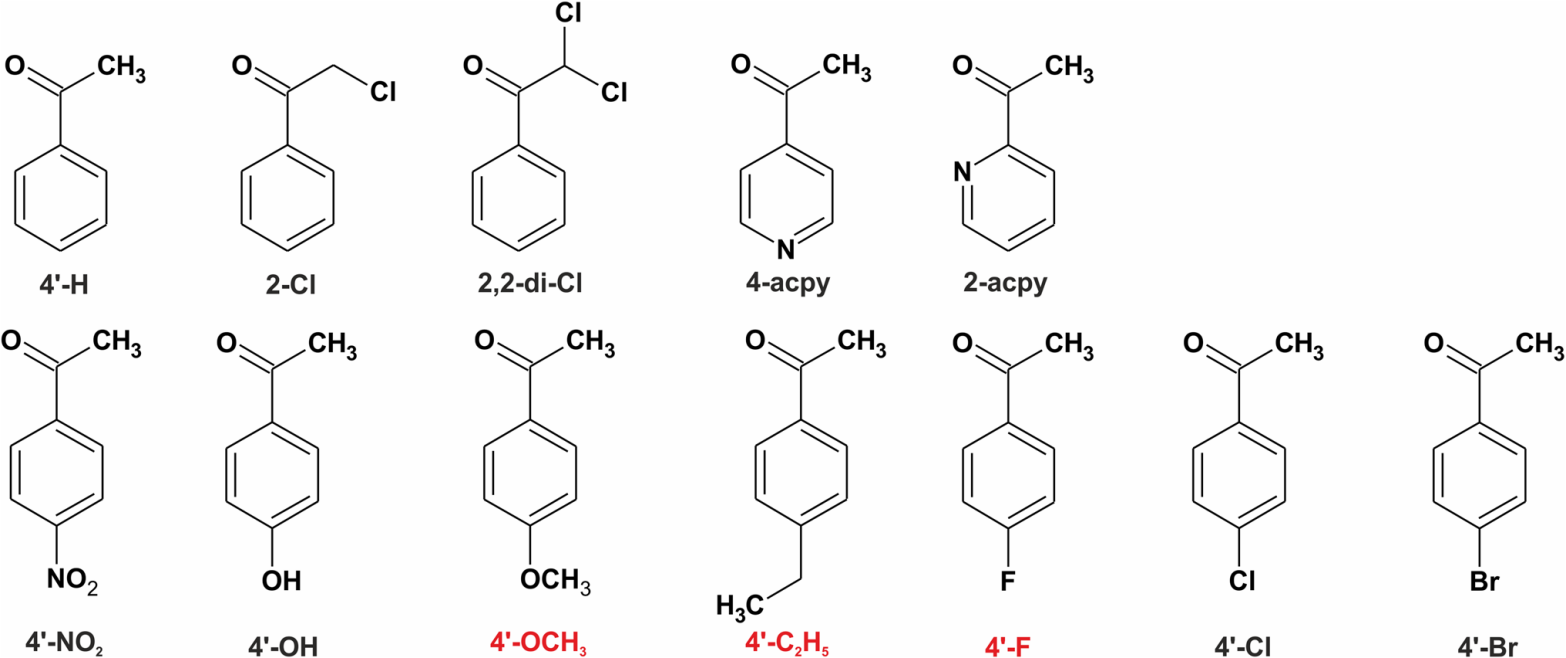
PEDH substrates used in the study. The *red labelled* substrates were used as an external validation set

The low pH cluster model was used to investigate kinetics and thermodynamics of the PEDH catalysed reductions of ketones to alcohols. The optimization of enzyme-ketone complexes (ES), transitions states (TS) as well as the enzyme-alcohol complexes (EP) were obtained for acetophenone and its structural analogues: 2,2-di-chloroacetophenone, 2-chloroacetophenone, 4′-nitroacetophenone, 4′-hydroxyacetophenone, 4′-methoxyacetophenone, 4′-ethylacetophenone, 4′-fluoroacetophenone, 4′-chloroacetophenone, 4′-bromoacetophenone, 2-acetyl- and 4-acetylpyridine (Fig. 3). As for 2-acetylpyridine two substrate binding poses were possible (either presenting N atom of the pyridine ring toward or away from keto group) two different variants were considered and averages of energetic and geometric descriptors were used in the statistical analyses.

## Results

### Thermodynamic and kinetic characterization of ketones reduction

The heats of ketones to alcohols reduction were measured with ITC [23–26]. The amount of heat (Q) involved in converting of *n* moles of selected substrate to the product is expresses by the equation:

$$\Delta H=\frac{Q}{n}$$

where ∆*H* is the total molar enthalpy (in kJ mol^−1^) for the tested reaction determined experimentally by ITC (average values Table 1, all results in Table S1 in the supplementary material). The number of moles of the selected substrate, which was converted into the product, was calculated on the basis of HPLC measurements.

**Table 1:** The average values of enthalpy: theoretically calculated and experimentally found, as well as an activation enthalpy of the tested reaction

Substrate	Δ*H*_*rITC*_ (kJ mol^−1^)	Δ*H*_*r*DFT_ (kJ mol^−1^)	ln k_cat_	Δ*G* ^#^ (kJ mol^−1^)
Acetophenone	−18.26	13.58	−0.31	103.89
4′-Hydroxyacetophenone	−9.63	19.47	−1.25	112.94
4′-Chloroacetophenone	−21.76	12.17	0.07	105.10
4′-Bromoacetophenone	−16.26	23.69	0.27	107.35
4′-Nitroacetophenone	−24.44	0.98	1.32	89.81
4-Acetylpyridine	−28.83	−3.62	1.88	89.02
2-Acetylpyridine	−22.57	10.32	1.70	99.95
2-Chloroacetophenone	−29.93	−5.06	0.34	99.08
2,2-Di-chloroacetophenone	−35.61	−14.30	1.51	86.58
4′-Methoxyacetophenone	n.d	20.87	n.d	113.48
4′-Fluoroacetophenone	n.d	15.20	n.d	110.19
4′-Ethylacetophenone	n.d	17.97	n.d	105.77

Δ*H*_*ITC*_ the enthalpy of the reaction of ketone with NADH measured with ITC, Δ*H*_*DFT*_ the enthalpy of the reaction calculated for cluster model, ln k_cat_ the average values of ln k_cat_ found in saturation conditions (1 mM substrate, 0.5 mM NADH), Δ*G* ^*#*^ the activation enthalpy obtained from DFT calculations

*n.d*. not determined

The initial rates for ketones reductions were established at the high substrate concentrations ensuring saturation of enzyme with substrates. The average values are collected in Table 1 while the full experimental results are available in the supplementary material (Table S2).

The theoretical values of thermodynamic functions corresponding to a transition state (marked by ^#^) or the overall energetic effect of a reaction were calculated on various levels of theory. The change in electronic energy was established for 6-31g(*d,p*) and 6-311+g(*2d,2p*) basis sets in the gas-phase as well as for 6-31g(*d,p*) in PCM solvent model (ε = 4). The solvent corrections established at 6-31g(*d,p*) level of theory were subsequently added to the further calculations (ΔE(6-311+g(*2d,2p*)) + PCM**)**. The vibrational corrections calculated at 6-31g(*d,p*) level of theory were added to the electronic energy established for 6-311+g(*2d,2p*). This resulted with ΔE(6-311+g(*2d,2p*)) + PCM with ZPE, enthalpy (*H*) and Gibbs free energy (*G*) corrections. Finally, all these values were corrected for dispersion interactions by single point D calculations [22].

This elaborate calculation scheme resulted with various theoretical descriptors which should, in theory, describe the reaction under study with an increasing accuracy. Our aim was to evaluate quantitatively, which of these corrections indeed introduce meaningful corrections to the phenomenon under study and assess if such descriptors can be used for prediction of the reaction energetic and kinetics.

The obtained experimental data enabled the validation of the theoretical model. A linear correlation analysis was used in order to obtained relation between enthalpy of the reaction (Δ*H*_*ITC*_) and energetics of reaction (i.e., energy difference between substrate and product) and geometries of transition state (in accordance with Hammond postulate). For explanation of the reaction rate (ln k_cat_) both the linear correlation and multivariate regression analysis was employed utilizing a range of energy values describing the height of the reaction barrier as well as various geometrical parameters describing advancement of the reaction (see “Correlation analysis” and further sections).

### Reaction mechanism—cluster model

**Fig. 4:**
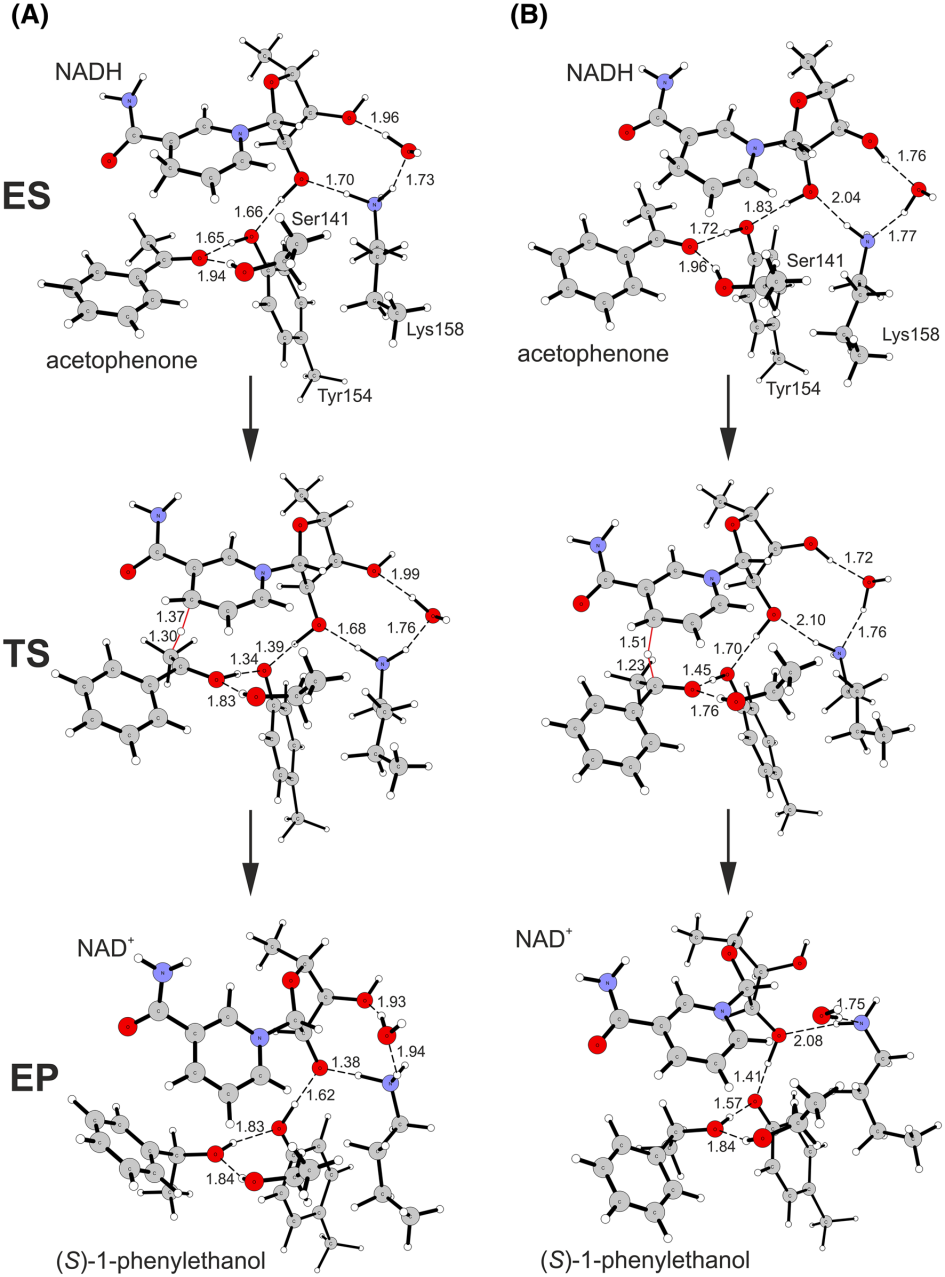
Reduction of acetophenone to (*S*)-1-phenylethanol catalysed by PEDH calculated with cluster model with B3LYP: **a** low pH model (protonated Lys158) and **b** high pH model (neutral Lys158). H-bond and crucial transition state distances were marked with *dashed lines* and their length provided in Å

The reaction of acetophenone reduction was investigated in two model pH conditions i.e., at the low pH (protonated Lys158) corresponding to the optimal conditions for ketone reduction and at the high pH (deprotonated Lys158) corresponding to the conditions optimal for alcohol oxidation (Fig. 4). In each case the modelling started with enzyme-acetophenone (ES) complex where acetophenone keto group formed two hydrogen bonds with the enzyme active site (Ser141 and Tyr154). The proton relay chain involved Tyr154, which was an H-bond acceptor to the O2′H group of the NADH ribose, which in turn formed H-bond with Lys158 NεH_3_/NεH_2_ group. In case of the low pH model Lys158 NεH_3_ was a donor of H-bond to H_2_O molecule. In case of the high pH model Lys158 NεH_2_ group was an acceptor of the H-bond from H_2_O molecule. Moreover, O3′H group of the NADH formed H-bond interaction with H_2_O, where H_2_O was a bond donor for low-pH models and a H-bond acceptor in the high-pH model. The transition state of the reduction reaction involved two elementary processes: (i) transfer of a hydride from NADH to the carbonyl carbon atom of the substrate and (ii) a shift of the proton in Tyr154-ketone H-bond from phenol group of Tyr to the oxygen atom of the reagent. Our calculations showed that in case of SDR these two processes proceed in concert. The independent preliminary calculations (conducted for acetophenone and 4′-OH derivative, data not shown) proved that the reduction process is not initiated by protonation of the keto group. If the keto group is protonated before the hydride starts to shifts towards carbon atom the proton spontaneously moves back to the Tyr154. The analysis of transitions state geometry shows that for the low pH model the TS is reached at an earlier stage, i.e., for a shorter C^NADH^–H bond (1.37 Å) and longer H–C^carb^ bond (1.30 Å) than in case of the high pH model (1.51 and 1.23 Å, respectively). Moreover, for the low pH model the H atom of the Tyr154 OH group is almost completely transferred to the carbonyl oxygen atom (*d*(C=O–H–OTyr) of 1.09 Å, *d*(H–OTyr) = 1.34 Å) while for the high pH model it is localized closer to TyrO^−^ (*d*(C=O–H–OTyr) of 1.45 Å and *d*(H–OTyr) of 1.05 Å). As a consequence also the next proton in the proton relay system is more advanced in the shift for the low pH model (*d*(Tyr-O–H–O2′-ryb) 1.40 Å) than in case of the high pH model (1.70 Å).

The analysis of the imaginary vibration associated with the transition state showed, that all three molecular motions (i.e., H^−^ transfer and the shifts of two protons) are coupled. Finally, for enzyme-alcohol complex (EP) the hydride and proton shifts are finalized and secondary alcohol is formed. The alcohol product is stabilized again by two H-bond interactions, one with Ser141 and the other with Tyr154. However, as is postulated for the high pH conditions, in the high pH model the Tyr154 is in the negative phenolic state, stabilized by H-bonds with the alcohol and ribose O2′H group. On the other hand for the low pH model Tyr154 is in a neutral form and the ribose O2′H group is deprotonated. Such form is however stabilized by a significant shortening of O–H–NεH_2_-Lys158 distance from 1.7 to 1.39 Å. It is expected that in the next step of the proton transfer cascade the ribose O^−^ group would get protonated and Lys158 reprotonated by H_3_O^+^ molecule from the solvent. Then again, this part of the reaction mechanism was beyond the scope of the conducted modelling.

**Fig. 5:**
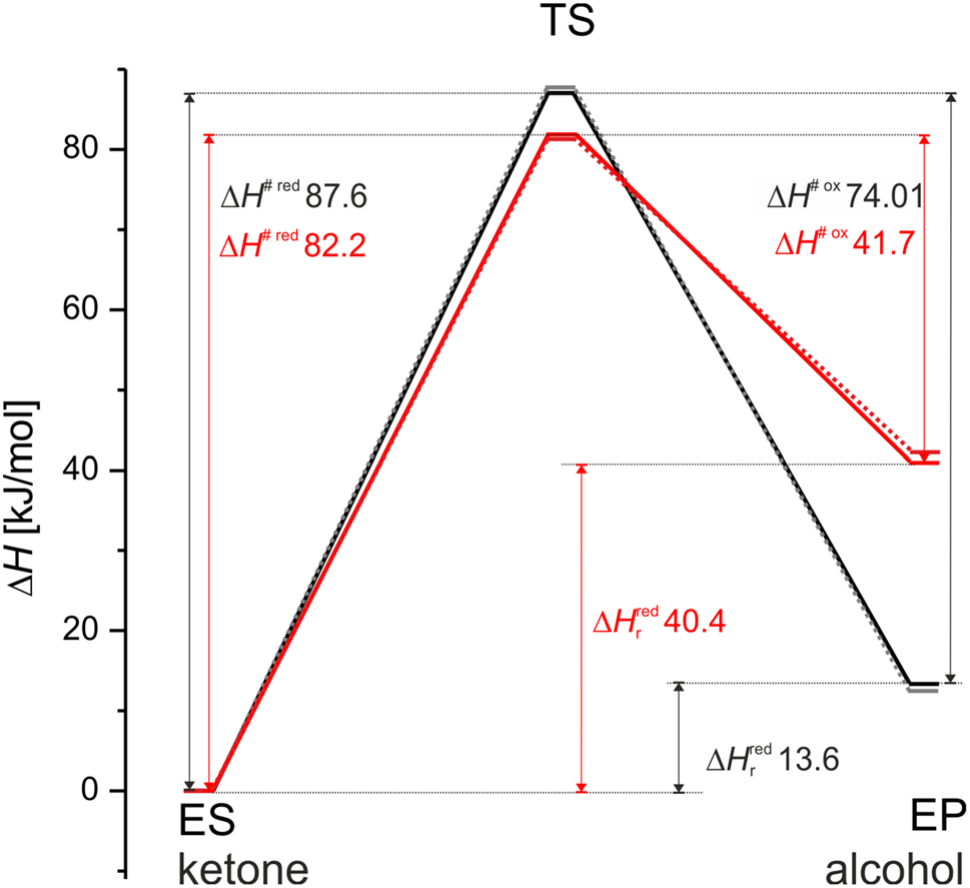
Comparison of enthalpy reaction profiles calculated for low pH (*black line*s) and high pH (*red line*s) models with B3LYP (*solid line*s) and X3LYP (*dotted line*s) functionals. Δ*H* ^# red^ and Δ*H* ^# ox^ are enthalpies of activation for reduction or oxidation process, respectively, while Δ*H*_r_^red^ are the reaction enthalpies for the reduction process

The analysis of reaction energetic profiles revealed that barriers between ES and TS are not sensitive to the protonation state, i.e., for both models similar energy barriers were observed (Table 2). On the other hand, the energetics of EP complex differed significantly. Due to the presence of the reactive tyrosyl anion in EP of the high pH model the system energy was elevated by approximately 30 kJ mol^−1^. As a result, the energy barrier for the alcohol dehydrogenation in the high pH is significantly lower compared to that in the low pH conditions (by 32.3 kJ mol^−1^). Moreover, the energy barriers associated with the oxidation of alcohol were always lower than barriers of ketone reduction, regardless of the model used. These facts are in qualitative agreement with the experimental data, which by and large validate our modelling approach [7]. Finally, the energies (and geometries) obtained with X3LYP functional yielded very similar results with similar tendencies comparing to those found with B3LYP functional. Therefore, as the DFT-D damping factors were not available for X3LYP, the rest of calculations were conducted with the latter one (Fig. 5).

**Table 2:** Energies of stationary points with various corrections calculated with B3LYP and X3LYP functionals for low and high pH cluster models

kJ mol^−1^		ΔE(6-31g(*d,p*))	ΔE(6-31g(*d,p*)) + PCM	ΔE(6-311+g(*2d,2p*))	ΔE(6-311+g(*2d,2p*)) + PCM	ΔE(6-311+g(*2d,2p*)) + PCM + ZPE	Δ*H* + PCM	Δ*G* + PCM	Δ*H* + PCM + D	Δ*G* + PCM + D
Low pH model										
B3LYP	TS	104.32	101.04	113.99	110.71	92.31	87.59	103.89	64.94	81.23
	EP	12.47	12.08	20.19	19.79	15.23	13.58	16.01	1.26	3.69
X3LYP	TS	102.8	99.7	111.9	108.7	93.0	87.9	105.4	n.d	n.d
	EP	11.4	10.5	19.6	18.7	14.5	12.8	15.9	n.d	n.d
High pH model										
B3LYP	TS	89.91	85.52	101.51	97.13	87.62	82.22	100.68	62.04	80.49
	EP	53.18	36.37	65.35	48.55	42.13	40.54	45.88	19.59	24.93
X3LYP	TS	89.7	85.0	101.1	96.4	86.6	81.4	98.5	n.d	n.d
	EP	50.7	33.8	63.2	46.3	39.8	38.3	42.1	n.d	n.d

The energies of ES of the particular model were always used as a reference (0 kJ mol^−1^)

*n.d*. not determined

The calculated energies of the barrier were in the range of the experimentally obtained values (E^a^ for acetophenone is in the range of 62–77.6 kJ mol^−1^—see Table S3 in the supplementary material), especially after vdW corrections were included in the calculations. However, it has to be remembered that the method used in the study was definitely not calibrated for delivering the exact experimental values (see below) and the whole aim of the study was to calibrate quantum chemical calculation with the experiment.

### The analysis of transition states for acetophenone derivatives

The analysis of transition state geometries for all studied acetophenone structural analogues delivered further interesting information on chemistry of the hydride transfer process in SDR (Table 3, see the supplementary material for figures of all stationary points). In all studied cases the proton relay system (Lys158–O2′-ribose–Tyr154–O=C) was involved in the protonation of the reactant carbonyl oxygen atom. All substrates could be divided into two general groups: those, for which at the TS the transfer of the H^+^ from Tyr154 to C=O group was already beyond the mid-point (i.e., is on the side of carbonyl oxygen) and those for which the proton was still closer to the Tyr154. 4′-OH, 4′-MeO, 4′-H (low pH), 4′-Et, 4′-F, 4′-Cl, 4′-Br and a *syn* conformer of 2-acpy belonged to the first group with the average C=O–H distance of 1.10 Å. For the rest of the studied compounds the proton transfer in TS was less advanced and the average C=O–H distance was 1.42 Å.

**Table 3:** The geometrical parameters of transition states for hydride transfer from NADH to acetophenone and its structural analogues and proton relay (i.e., Lys158-ribose-Tyr154–O=C of the reactant)

Model	C^NADH^–H	H–C	∡C^NADH^HC	C=O–H-OTyr	H–OTyr	Tyr-O–H-O2′-ryb	H–O2′-ryb	ryb-O2′–H-NH_2_-Lys	H–NH_2_-Lys
4′-H high pH X3LYP	1.508	1.233	158.305	1.441	1.051	1.694	0.991	2.086	1.022
4′-H low pH X3LYP	1.372	1.297	161.462	1.098	1.343	1.467	1.044	1.814	1.043
4′-H high pH	1.512	1.234	158.409	1.447	1.051	1.702	0.991	2.097	1.023
4′-H low pH	1.373	1.303	162.073	1.097	1.345	1.397	1.069	1.679	1.058
4′-OH	1.390	1.289	162.019	1.074	1.394	1.449	1.052	1.801	1.045
4′-Cl	1.376	1.303	162.052	1.112	1.321	1.413	1.062	1.682	1.057
4′-Br	1.383	1.297	162.166	1.116	1.315	1.410	1.065	1.660	1.060
4′-NO_2_	1.478	1.249	159.925	1.324	1.110	1.509	1.026	1.714	1.052
4-acpy	1.489	1.246	158.947	1.400	1.071	1.534	1.020	1.701	1.052
2-Cl	1.442	1.276	160.020	1.447	1.048	1.543	1.017	1.721	1.052
2,2-di-Cl	1.431	1.295	157.629	1.493	1.033	1.554	1.015	1.728	1.051
4′-MeO	1.394	1.287	165.619	1.069	1.405	1.442	1.054	1.798	1.045
4′-F	1.379	1.299	162.195	1.104	1.334	1.404	1.066	1.683	1.058
4′-Et	1.377	1.299	162.498	1.079	1.380	1.375	1.080	1.666	1.060
2-acpy *syn*	1.376	1.312	158.056	1.125	1.294	1.402	1.066	1.659	1.060
2-acpy *anti*	1.478	1.249	159.821	1.300	1.124	1.497	1.031	1.695	1.055

All distances are provided in Å and the angle between C^NADH^–H–C^subtrate^ in °

Based on Hammond postulate one would expect earlier TS (more reactant-like, less advanced hydride shift, i.e., longer C^NADH^–H distance compared to the H–C distance) for those reactions which are more exothermic [27]. Such effect is clearly visible in case of the low and high pH models of 4′-H where less endothermic low pH mechanism results with the TS at shorter C^NADH^–H distance (1.372 Å) when compared to the high pH model (1.508 Å). This effect is even more apparent if an alcohol product is assumed as a reactant state for the alcohol oxidation process. Then the high pH process becomes ‘more exothermic’ than the low pH and, consequently, the TS is clearly more ‘reactant-like’ in accordance with the Hammond postulate. However, the general correlation for all studied compounds is relatively weak and unconvincing (R^2^ < 0.5). On the other hand, the proton shift to the carbonyl group concomitant with a hydride transfer turned out to be much more indicative descriptor of the TS advancement and consistent with Hammond postulate (see correlation analysis below).

The analysis of transition state barrier Gibbs free energies (Δ*G* ^#^) clearly indicates that the introduction of the electron withdrawing group to the acetophenone core lowers the energy barrier of hydride transfer. This effect was especially visible for 2,2-di-Cl (relative ΔΔ*G* ^#^ −17.30 kJ mol^−1^, where Δ*G* ^#^ of acetophenone was used as a reference) and to a lesser extent for 2-Cl (ΔΔ*G* ^#^ −4.81 kJ mol^−1^), which exerts closest induction effect on the carbon atom directly adjacent to the carbonyl atom that is being reduced during the H^−^ transfer. Similar effect was also observed for substituents/ring modifications that were introduced into the aromatic ring although the electron withdrawing shift had to be exerted on the carbonyl atom indirectly through the aromatic ring system. The highest stabilization of the reaction barrier was calculated for 4-acpy and 4′-NO_2_ (ΔΔ*G* ^#^ −14.87 and −14.08 kJ mol^−1^, respectively). On the other hand for substituents that increased the electron density of the ring system (either through induction or resonance effect) such as 4-Et, 4-MeO and 4-OH the relative increase of Δ*G* ^#^ was observed in the range of 1.88–9.6 kJ mol^−1^. Interestingly, the substitution of acetophenone with 4′-halogene atoms also resulted with increase of the Δ*G* ^#^ with 4′-Cl exhibiting the lowest increase of 1.22 kJ mol^−1^.

### Correlation analysis

The obtained theoretical descriptors were correlated with experimental values: Δ*H*_r_ (ITC) and ln k_cat_ (see the supplementary Excel spreadsheet for all data). The analysis was initiated by calculation of the Pearson linear correlations between descriptors and experimental variables (Table 4).

**Table 4:** Results of correlation analysis between theoretical descriptors and experimental heat of reaction Δ*H*_r_ and natural logarithm of reaction rate (ln k_cat_)

Experimental value	Δ*H*_r_		ln k_cat_		ln k_cat_ without 2-acpy	
Descriptor	R	R^2^	R	R^2^	R	R^2^
ΔE(6-31g(*d,p*))	0.70	0.50	−0.82	0.67	−0.89	0.79
ΔE(6-31g(*d,p*)) + PCM	0.85	0.72	−0.82	0.68	−0.90	0.82
ΔE(6-311+g(*2d,2p*))	**0.96**	**0.93**	−0.73	0.54	−0.82	0.67
ΔE(6-311+g(*2d,2p*)) + PCM	**0.95**	**0.91**	−0.77	0.60	−0.87	0.76
ΔE(6-311+g(*2d,2p*)) + PCM + ZPE	0.91	0.83	−0.83	0.69	−0.91	0.82
Δ*H* + PCM	**0.93**	**0.87**	−0.82	0.66	−0.89	0.80
Δ*G* + PCM	0.76	0.57	−**0.88**	**0.78**	−**0.94**	**0.89**
ΔE(6-31g(*d,p*)) + vdW	0.28	0.08	−0.60	0.37	−0.67	0.46
ΔE(6-31g(*d,p*)) + PCM + vdW	0.41	0.17	−0.66	0.43	−0.74	0.55
ΔE(6-311+g(*2d,2p*)) + vdW	0.61	0.37	−0.51	0.26	−0.60	0.36
ΔE(6-311+g(*2d,2p*)) + PCM + vdW	0.74	0.55	−0.60	0.36	−0.69	0.48
ΔE(6-311+g(*2d,2p*)) + PCM + ZPE + vdW	0.81	0.66	−0.61	0.37	−0.68	0.47
Δ*H* + PCM + vdW	0.78	0.61	−0.59	0.35	−0.67	0.45
Δ*G* + PCM + vdW	**0.97**	**0.95**	−0.71	0.50	−0.77	0.60
C^NADH^–H	−0.66	0.44	0.76	0.58	0.79	0.63
H–C	0.39	0.16	−0.59	0.34	−0.61	0.37
∡C^NADH^HC	0.85	0.73	−0.85	0.71	−0.83	0.69
C=O–H–OTyr	−**0.93**	**0.86**	0.73	0.53	0.8	0.64
H–OTyr	**0.93**	**0.86**	−0.8	0.64	−0.85	0.73
Tyr-O–H–O2′-ryb	−0.81	0.66	0.59	0.35	0.68	0.46
H–O2′-ryb	0.82	0.67	−0.62	0.38	−0.7	0.49
ryb-O2′–H−NH_2_-Lys	0.04	0.00	−0.47	0.22	−0.42	0.18
H–NH_2_-Lys	0.14	0.02	0.26	0.07	0.19	0.04

The R and R^2^ for ln k_cat_ were calculated for the whole dataset (ln k_cat_) and for the data set with 2-acpy excluded as an outlier (ln k_cat_ without 2-acpy)

The best correlations were marked with bold

### Prediction of Δ*H*_r_

**Fig. 6:**
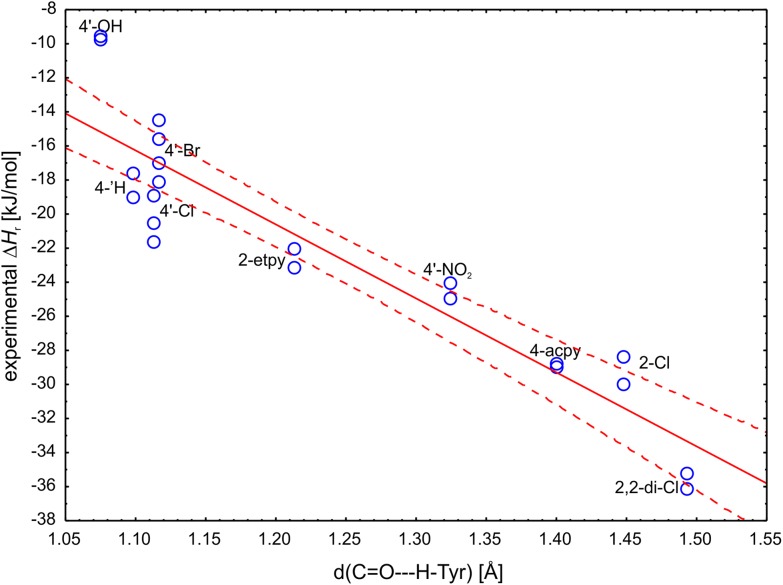
Correlation plot of the experimental reaction enthalpy Δ*H*_r_ with C=O–H–OTyr distance (R^2^ = 0.8573). The *solid line* represents linear fit while *dashed line* represents 95% confidence range

As was already mentioned the progress of the proton shift in TS turned out to be in accordance with the Hammond postulate. The correlation between *d*(C=O–H–OTyr) and the experimental Δ*H*_r_ (R = −0.9259) explained 85% of the observed variance and only 4′-OH compound was positioned outside of the 95% confidence boundaries (Fig. 6). This correlation indicated that the more exothermic the reduction of ketone the less advance is the shift of the proton in the transition state toward the reactant (i.e., the earlier, more reactant-like TS). As a result one can use this geometric descriptor to predict the reaction energetics (with a standard estimation error of 2.83 kJ mol^−1^). To a lesser extent this correlation is also visible in the next proton relay between ribose 2′-OH and Tyr but only 66% of the variability can be explained by these distances.

$$\Delta H\,_{{\text{r}}} = \, - {\text{42}}.{\text{425 }}\left( { \pm {\text{4}}.0{\text{64}}} \right)\,d({\text{C}} = {\text{O}}{-}{\text{H}}{-}{\text{OTyr}})\, +{\text{31}}.{\text{5 }}\left( { \pm {\text{5}}.0{\text{49}}} \right){\text{ }}[{\text{kJ}}\,{\text{mol}}^{{ - {\text{1}}}} ].$$

$${\text{n}}\, = \,{\text{21}},{\text{ R}}^{{\text{2}}} \, = \,0.{\text{8573}},{\text{ R}}^{{\text{2}}} _{{{\text{corr}}}} \, = \,0.{\text{8498}},{\text{ F}}\, = \,{\text{114}}.{\text{18}},{\text{ p}}\, = \,{\text{1}}.{\text{79}}\, \times \,{\text{1}}0^{{ - {\text{9}}}} ,{\text{error of estimation}}\, = \,{\text{2}}.{\text{832}}.$$

**Fig. 7:**
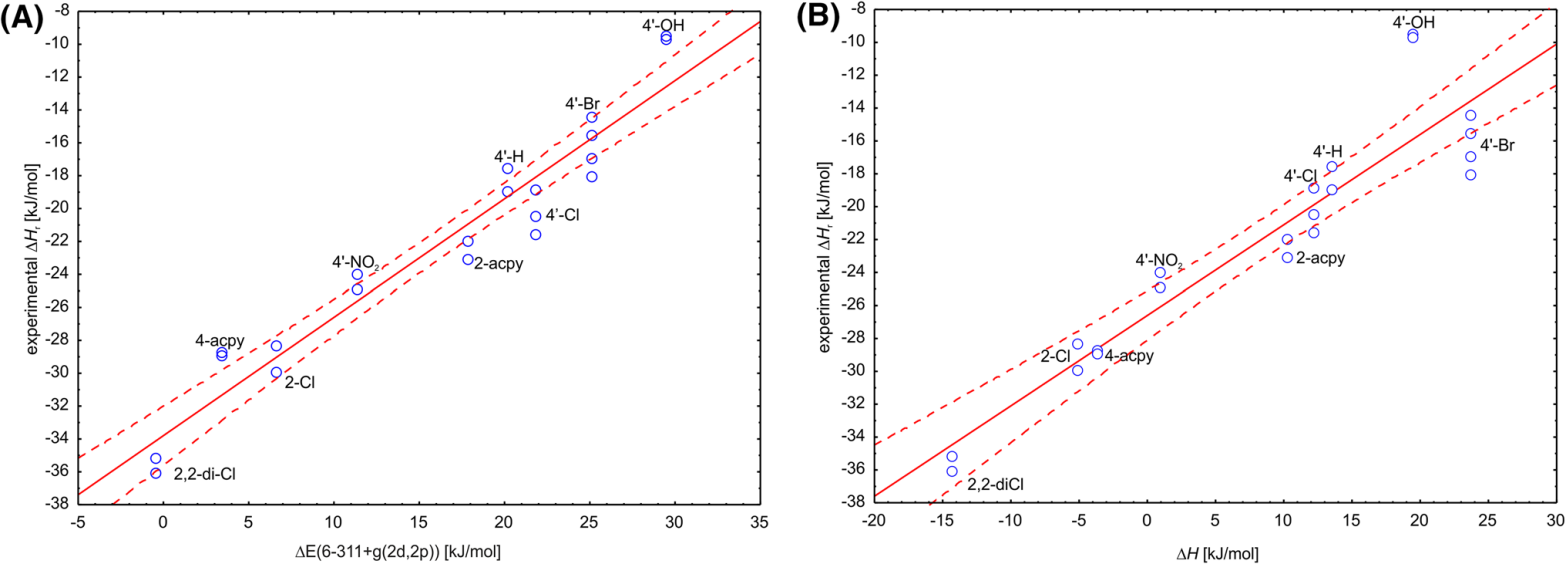
Correlation plot of the experimental reaction enthalpy ∆*H*_r_ with energetic descriptors calculated with cluster models: **a** ∆E(6-311+g(*2d,2p*)) (R^2^ = 0.9292) or **b** ∆*H* (R^2^ = 0.8713). The *solid line* represents a linear fit while *dashed line* represents 95% confidence range

Moreover, the correlation analysis showed that the best descriptor predicting Δ*H*_r_ is ΔE(6-311+g(*2d,2p*)) explaining 93% of the observed variation (R^2^ = 0.9292) followed by ΔE(6-311+g(*2d,2p*)) + PCM and Δ*H* which explained 91 and 87% of the observed experimental variation (Table 4). As a result the experimental value of Δ*H*_r_ can be predicted with a very good accuracy according to the following formulas (Fig. 7):

$$\Delta H_{{\text{r}}} = 0.{\text{7196}}\, \left( { \pm 0.0{\text{456}}} \right)\,\Delta {\text{E}}\left( {{\text{6}} - {\text{311}}\, + \,{\text{g}}\left( {2d,2p} \right)} \right) - \,{\text{33}}.{\text{796 }}\left( { \pm 0.{\text{86}}} \right) {\text{ }}[{\text{kJ}}\,{\text{mol}}^{{ - {\text{1}}}} ].$$

$${\text{n}}\, = \,{\text{21}},\;{\text{R}}^{{\text{2}}} \, = \,0.{\text{9292}},\;{\text{R}}^{{\text{2}}} _{{{\text{corr}}}} = \,0.{\text{9255}},{\text{ F}}\, = \,{\text{249}}.{\text{53}},{\text{ p}}\, = \,{\text{2}}.{\text{21}} \times {\text{1}}0^{{ - \,{\text{12}}}} ,\;{\text{error of estimation}}\, = \,{\text{1}}.{\text{994}}.$$

$$\Delta H_{{\text{r}}} = {\text{ }}0.{\text{5499 }}\left( { \pm 0.0{\text{485}}} \right)\,\Delta H - {\text{26}}.{\text{6}}0{\text{3 }}\left( { \pm 0.{\text{711}}} \right){\text{ }}[{\text{kJ}}\,{\text{mol}}^{{ - {\text{1}}}} ].$$

$${\text{n}}\, = \,{\text{21}},{\text{ R}}^{{\text{2}}} = \,0.{\text{8713}},\;{\text{R}}^{{\text{2}}} _{{{\text{corr}}}} = \,0.{\text{8645}},{\text{ F}}\, = \,{\text{128}}.{\text{57}},{\text{ p}}\, = \,{\text{6}}.{\text{7}}0 \times {\text{1}}0^{{ - \,{\text{1}}0}} ,{\text{error of estimation}}\, = \,{\text{2}}.{\text{69}}0.$$

The fact that ΔE(6-311+g(*2d,2p*)) descriptor, which carries no information on vibrational energy of the model system, turned out to be better than the Δ*H* (corrected with solvent corrections) suggested that introduction of solvent corrections may have slightly decreased the correlation. Indeed, if the Δ*H* without solvent corrections is used as a descriptor (Δ*H* ^no solvent^) it can explain 90% of the experimental variation (R^2^ = 0.8956). It seems that most of the variations between the substrates is well described by differences of the electronic energy and therefore such calculations (on the TZVP basis set level of theory) are enough for decent prediction of the system thermodynamics.

The introduction of vdW corrections generally decreased the correlation quality with Δ*H*_r_. Unexpectedly, the best correlations was observed with Δ*G*+vdW which could explained almost 95% of the experimental variation (R^2^ 0.9460—see Fig. S1 of the supplementary material). However, as Δ*H*+vdW exhibited lower correlation with the experimental Δ*H*_r_ than the Δ*H* not corrected by vdW, we must assume that this effect is most probably due to some compensation of errors.

### Prediction of ln k_cat_

The prediction of reaction rate proved to be more challenging than the reaction enthalpy. First of all the results for 2-acpy turned out to be outliers in every analysis (for each of the isomers and for an average as well). Therefore, 2-acpy was excluded from further analyses.

The correlation analysis revealed that the best descriptor for prediction of ln k_cat_ is Δ*G* ^#^ (R^2^ = 0.8866) which explained 89% of the experimental variability followed by ΔE(6-311+g(*2d,2p*)) + PCM + ZPE and ΔE(6-31g(*d.p*)) + PCM (R^2^ = 0.8208 and 0.8159, respectively).

Similarly as in case of Δ*H*_r_, introduction of vdW correction decreased the quality of correlation with the experiment and the best results were observed for Δ*G* ^#^+vdW (R^2^ = 0.5981).

**Fig. 8:**
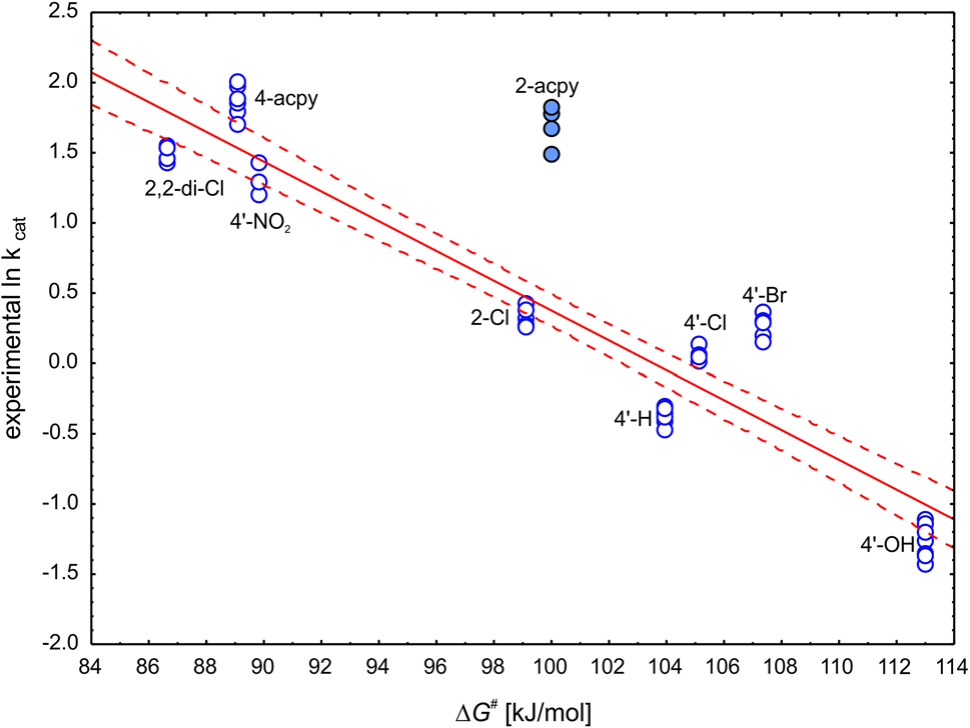
Correlation plot of the ln k_cat_ with ∆*G* ^#^ (R^2^ = 0.8866). The solid line represents linear fit while dashed line represents 95% confidence range. The outlier 2-acpy (full *circles*) was excluded from the correlation

Based on these results the following regression models can be used to predict ln k_cat_ of reduction of 4′- and 2- substituted structural analogues of acetophenone (Fig. 8):

$${\text{ln k}}_{{{\text{cat}}}} \, = \, - 0.{\text{1}}0{\text{61}}\,\left( { \pm 0.00{\text{61}}} \right)\,\Delta G^{{\# \,}} + \,{\text{1}}0.{\text{98 }}\left( { \pm \,0.{\text{61}}} \right)[{\text{kJ}}~{\text{mol}}^{{ - {\text{1}}}} ].\,$$

$${\text{n}}\, = \,{\text{41}},{\text{ R}}^{{\text{2}}} = \,0.{\text{8866}},\;{\text{R}}^{{\text{2}}} _{{{\text{corr}}}} = \,0.{\text{8837}},{\text{ F}}\, = \,{\text{3}}0{\text{4}}.{\text{9}}0,{\text{ p}} = {\text{4}}.0{\text{8}}\, \times \,{\text{1}}0^{{ - {\text{2}}0}} ,\;{\text{estimation error}}\, = \,0.{\text{363}}.$$

The correlations with geometrical descriptors derived from transition state geometries turned out to be inferior to that with energetic descriptors, and the highest R^2^ (0.7246) was observed for *d*(H–OTyr). Most of them were linearly correlated with Δ*G* ^#^ and they did not introduce any non-redundant information. As a result, most of them could not be used to supplement Δ*G* ^#^ in prediction of the ln k_cat_. However, three descriptors (*d*(H-C), *d*(ryb-O–H-NH_2_-Lys) and *d*(H–NH_2_-Lys), two latter linearly dependent from each other) were not correlated with Δ*G* ^#^ and enabled construction of a more complex MLR that proved to be slightly more precise in prediction of ln k_cat_:

$${\text{ln k}}_{{{\text{cat}}}} \, = \, - 0.0{\text{52 }}\left( { \pm \,0.00{\text{8}}} \right)\,\Delta G^{\# } \, \, - {\text{5}}.{\text{78 }}\left( { \pm {\text{1}}} \right) d\left( {{\text{ryb-O}}{-}{\text{H-}} {\text{NH}}_{{\text{2}}}{\text{-Lys}}} \right) - {\text{9}}.{\text{363 }}\left( { \pm {\text{2}}.{\text{516}}} \right) \,d\left( {{\text{H}}{-}{\text{C}}} \right)\, + \,{\text{3}}0.{\text{99 }}\left( { \pm \,{\text{3}}.{\text{73}}} \right).$$

$${\text{n}}\, = \,{\text{41}},{\text{ R}}^{{\text{2}}} \, = \,0.{\text{9439}},{\text{ corr}}.{\text{ R}}^{{\text{2}}} \, = \,0.{\text{9394}},\;{\text{F}}\, = \,{\text{2}}0{\text{7}}.{\text{62}},{\text{ p}}\, = \,{\text{3}}.{\text{43}}\, \times \,{\text{1}}0^{{ - {\text{23}}}} ,{\text{ estimation error}}\, = \,0.{\text{397}}.$$

The analysis of MLR β coefficients showed that the most important descriptor is the Δ*G* ^#^ (−0.77) followed by *d*(ryb-O–H-NH_2_-Lys) (β = −0.24) and *d*(H–CO) (β = −0.19). The scatter plot presenting quality of the prediction is shown in Fig. S2 of the supplementary material.

Moreover, such approach enabled prediction of the ln k_cat_ including 2-acpy with corr. R^2^ of 0.8753 (see supplementary material for the equation and Fig. S3 for the correlation plot).

### Validation

We have decided to test the obtained relations in prediction of the equilibria in the real reactor system. In order to be able to do this we had to extend the validation set and calculate Δ*H*_r_ for three additional acetophenone analogues (4′-MeO, 4′-F, 4′-Et), which were not experimentally characterized in this study by ITC. However, for these compounds experimental estimates of the log *K* were previously established in the whole-cell reactor system. In those reactor tests the ketones were reduced in the presence of sacrificial co-substrate, isopropanol, which was used in the recovery of NADH. In such systems the NAD^+^ produced during ketone reduction is converted back to NADH in a reverse reaction (catalysed by the same SDR) with isopropanol, yielding acetone in the system. As a result, the equilibrium constant contains not only the concentration of the acetophenone derivative and the respective alcohols, but also isopropanol (introduced to reactor in a significant excess to the other reagents) as well as the acetone (with final concentration slightly lower than the alcohol product due to initial pool of NADH in bacteria).

As the reactions were carried out for an extended period of time (up to 10 days) and were subjected to multiple HPLC sampling, in case of the longer reactions the acetone produced in the mixture evaporated from the system. Such phenomenon was indicated by the increased sum of substrate and product concentrations (due to decrease of the total reactor volume). Although in calculations of the equilibrium constant the ‘real’ concentrations of reagents were re-calculated based on the initial concentration of the substrate and relative concentrations of reagents at the end of reactor run, the final equilibrium might have been shifted toward the alcohol products due to the evaporation of the acetone from the system. The points for which the decrease of the final reactor volume was observed were marked in blue on the correlation plots in order to indicate a possible bias associated with such results. As mentioned earlier we decided to check how our theoretical descriptors perform in prediction of log *K* of the real reactor system for all reactor runs, not excluding those for which the potential overestimation of the log *K* was possible.

**Fig. 9:**
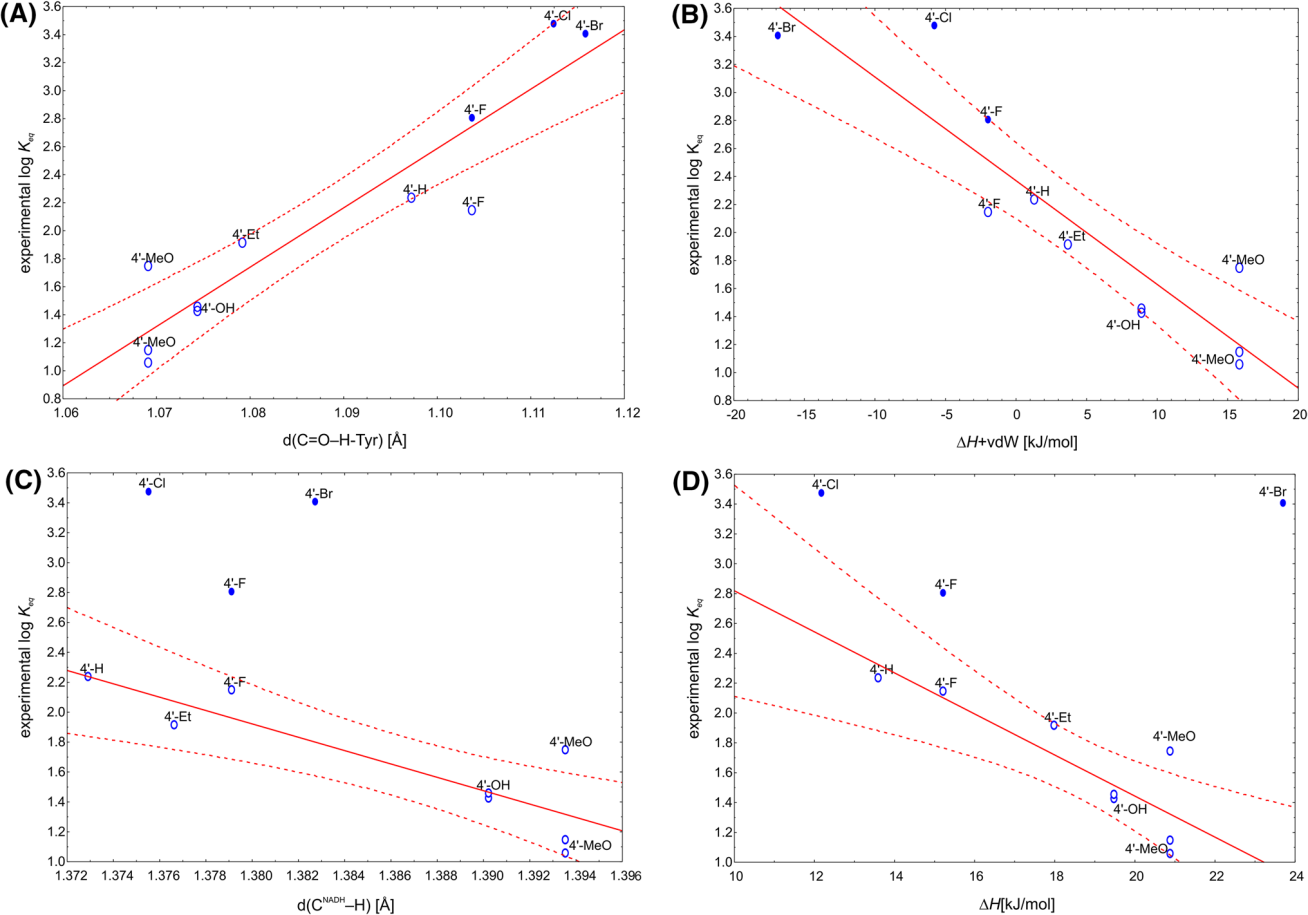
Correlation plot of the log *K*_*eq*_ with: (A) *d*(C=O–H-Tyr) (R^2^ = 0.8728) and (B) ∆*H* + vdW (R^2^ = 0.8277) for the whole data set of the experimentally determined *K*_*eq*_; (C) *d*(CNADH–H) (R^2^ = 0.7494) and (D) ∆*H* (R^2^ = 0.7502) for the data set with excluded values, which were potentially overestimated (full ovals). The *solid line* represents linear fit while *dashed line* represents 95% confidence range

Surprisingly, the correlation analysis with all theoretical descriptors showed that experimental log *K* can be well predated by Δ*H* + vdW (R^2^ = 0.8277) or ΔE + ZPE + vdW (R^2^ = 0.8259) or even better by geometrical descriptors *d*(C=O–H-Tyr) and *d*(Tyr-O–H) (R^2^ = 0.8728 and 0.8566, respectively). In case the overestimated log *K* (for 4′-Br, 4′-F and 4′-Cl) were eliminated from the data set the best correlations were observed for ΔE(6–31 + g(*d,p*)), ΔE(6–31 + g(*d,p*))+PCM, ΔE(6-311+g(*2d,2p*)) + PCM (R^2^ = 0.78) (Fig. 9) as well as Δ*H* (R^2^ = 0.7502). The best geometrical descriptors were *d*(C^NADH^ **–**H) and *d*(Tyr-O**–**H) explaining 75 and 73% of experimental variability. However, as the experimental error of *K*_*eq*_ determination was very high the particular preference for a given descriptor may be fortuitous. Nevertheless, as obtained trends agree with physicochemical interpretation of the process (see “Discussion” section) it can be assumed that our approach provides a quantitative method of prediction of equilibrium point for the real (i.e., industrial type) process of chiral alcohol synthesis and validation of the utility of our modelling approach.

## Discussion

Despite the fact that mechanism of the SDR enzymes has been extensively studied for years by both experimental and theoretical approaches there still are plenty ambiguities about the process. However, the sequence analyses, enzyme mutation and kinetic studies unequivocally confirmed that Ser, Tyr and Lys triad is essential to the catalytic mechanism [15, 28–33]. The structural studies also confirmed involvement of the conserved water molecule and Asn in the proton relay system [15, 28, 34, 35]. Most of the studies point also at Tyr residue as the acid/base catalysts responsible for protonation of the carbonyl group or deprotonation of the alcohol hydroxyl, although if Tyr is mutated to Phe this role can be conducted by Ser [28]. It was also suggested that positive charge of Lys may stabilize phenolate form of Tyr [31, 36]. Furthermore, plenty of studies have been aimed at discerning the protonation of various residues at different pH and the intricate working of the H-bonding network [36–38]. This issue is indeed a bit complicated especially if studied for oxidation process, when the pH dependent protonation state of Tyr influences the alcohol binding and the energy barrier of the oxidation process. However, in our case since we started with ketone as a substrate it was assumed that Tyr was in the protonated state, regardless of the pH. This was due to presence of the favourite hydrogen bonding between substrate carbonyl group and TyrOH and known fact of a proton transfer concomitant with a hydride transfer.

Such protonation was also confirmed in previous docking studies both for PEDH and Hped [8, 39]. Moreover, our QM calculations (data not shown) demonstrated, that if TyrO^−^/Lys-NH_3_^+^ model is formed with ketone, TyrO^−^ spontaneously strips proton from O2′ ribose and proton from Lys-NH_3_^+^ is transferred on O2′ atom, resulting with formation of TyrOH/Lys-NH_2_ system. As in such model the ketone carbonyl is stabilized only by H-bond with Ser141 and one of the NH_2_ protons is not involved in the H-bonding network the overall energetics is higher than respective high pH model, that has properly arranged H-bonding network. Therefore, despite the fact that it was predicted that Tyr and Lys are being protonated/deprotonated in concert (i.e., as a single titratable site) and significant fraction of Tyr seems to remain deprotonated while Lys appears to be protonated in the whole range of pH [38] we decided to model deprotonation of the whole proton relay only at the Lys158. The change of protonation of Lys158 had a very small effect (5 kJ mol^−1^) on the height of the energy barrier of the ketone reduction, but a very significant effect on the overall energetics of the process. For the high pH model the EP enthalpy was significantly elevated (Δ*H* of 26.6 kJ mol^−1^) compared to the EP of the low pH model. This resulted with the lower barrier for alcohol oxidation in high pH. Moreover, in case of the high pH model for EP the Tyr154 is indeed in the phenolic, charged state as postulated by many authors, stabilized by H-bonds with alcohol product and O2′ hydroxyl of ribose. These findings indicate that pH influences the kinetics of the oxidation process and the reaction equilibrium of the reduction process. Moreover, our results do not confirm a hypothesis that the protonation of Lys158 stabilizes phenolic state of Tyr154, as we observed formation of the TyrO^−^ only in case of deprotonated (i.e., neutral) Lys. In case of the low pH model the proton transfer turns out to be more advanced, i.e. the proton from ribose hydroxyl group is already positioned on Tyr, while alkoxide, stabilized by two H-bonds, is formed on the ribose O2′. This result would suggest that indeed lower pH and higher ‘concentration’ of protons in the relay system shift the equilibrium of the reduction process by slowing down the oxidation (i.e., by preventing the formation of the reactive TyrO^−^ species).

The analysis of transition states also confirmed the concomitant nature of the hydride transfer and proton shift from the Tyr154 to ketone carboxyl group. However, in our model for all studied compounds these processes were combined with concomitant transfer of the proton from ribose to Tyr. Therefore, at low pH all three elementary processes seem to be associated with just one energy barrier. This effect is not observed in case of the high pH model, i.e., only Tyr proton and hydride transfer are coupled, while motion of the proton of the O2′ hydroxyl is only slightly associated with imaginary frequency of the hydride transfer. We therefore assume that, indeed, positive charge of NεH_3_ group lowers the pK_a_ not of Tyr itself, as was suggested previously [37], but of the ribose O2′ hydroxyl, which is then able to transfer proton to Tyr more readily. Our results also confirm suggestion of Koumanov et al. about the key role of O2′ hydroxyl as a switch responsible for guiding direction of proton flow in the proton relay system, because the oxidation of alcohol after H^−^ transfer and protonation of the Tyr154 would require rotation of O2′ hydroxyl group toward Lys158 [38].

Up to date the hydride transfer processes, especially in case of LADH, were extensively studied theoretically [40–44]. However, the LADH is a medium-chain, Zn containing MDR, and despite several mechanistic similarities to SDR, it differs in some crucial details (e.g., deprotonation of alcohol is frequently postulated to proceed before hydride transfer). Moreover, the focus of these studies was mainly on the kinetic isotopic effect associated with H^−^ transfer. Surprisingly, the theoretical studies of SDRs were much less frequent [30, 36–38, 45, 46] and we were unable to find works that investigated the mechanism with DFT methods. Therefore, despite the abundant literature on SDR, it seems that our paper might be one of the first, that addresses the mechanism with DFT calculations. Our relatively simple QM-cluster model turned out to be surprisingly robust in prediction of process energetics. Of course the absolute values obtained in calculations differed from those measured in the experiment but the observed correlations enabled excellent prediction of both reaction enthalpy as well as a good prediction of reaction kinetics. The latter fact is especially notable, as also in case of SDR the KIE is an important factor contributing to the observed kinetics [47]. Our calculations confirmed that molecular factors stabilizing transition state are also stabilizing ketone form (thus decreasing enthalpy of reaction), a fact we previously postulated based on simple thermochemical calculations and artificial neural network modelling of reactor kinetics [8]. The influence of electron withdrawing groups on kinetics of SDR was already quantitatively studied with rat liver 3α-hydroxysteroid dehydrogenase for a series of p-substituted acetophenone structural analogues [48] and our conclusions are in general agreement with the results presented there. However, our calculation scheme is independent of the availability of Hammett σ, as demonstrated by incorporation of 4-acpy as well as 2-Cl and 2,2-di-Cl derivatives. It turned out that the experiment-calibrated theoretical prediction method can use solely electronic energy for good prediction of experimental Δ*H*_*r*_ and that vdW corrections do not improve the observed correlation. We were also able to correlate the reaction energetics with the degree of the proton transfer in TS, which is in quantitative agreement with Hammond postulate. The geometric features of the proton transfer and hydride shift were also used for MLR model which to some degree enhanced prediction of kinetics (ln k_cat_) obtained solely from Δ*G* ^#^.

To validate our prediction scheme we used equilibrium constant established from real life reactor systems, which exhibited high experimental error. However, despite that significant discrepancies in values of experimentally determined *K*_*eq*_ (due to more complex experimental setup and long-time of the experiments), we were able to obtain satisfactory degree of prediction. It seems that higher utility of vdW corrected descriptors in that case might have been an artefact, so further studies specially aimed at validation of our claims are required.

## Conclusions

The observed reaction rates were obtained in the uniformed spectrophotometric assay. The heats of the reaction were measured using calorimetry methods (ITC). Taking these experimental values we have tested both energetic descriptors obtained at different level of theory and geometric parameters as predictors of reaction rates and reaction enthalpies. We have shown that the magnitude of the reaction rate and reaction energetics can be fairly well predicted based on relatively simple DFT calculations. The validity of this approach was checked in prediction of end points for the real reactor tests which were modelling the industrial batch processes. Moreover, we believe that as alcohol dehydrogenases form a very mechanistically uniform class of biocatalysts our system can be used to predict the kinetics and thermodynamics of any enzyme of this SDR class.

## Electronic supplementary material

Below is the link to the electronic supplementary material.

Supplementary material 1 (XLSX 27 KB)

Supplementary material 2 (DOCX 2206 KB)
