# Supplementary material for: DFT-based prediction of reactivity of short-chain alcohol dehydrogenase
Source: J Comput Aided Mol Des. 2017 May 26;31(6):587–602. doi: 10.1007/s10822-017-0026-5 (PMC5487757; doi:10.1007/s10822-017-0026-5)
Supplement: Supplementary file 2 — Supplementary material 2 (DOCX 2206 KB) [file 10822_2017_26_MOESM2_ESM.docx]

Supplementary material

**DFT-based prediction of reactivity of short-chain alcohol dehydrogenase**

I. Stawoska^1^, A. Dudzik^2^, M. Wasylewski^2^, M. Jemioła-Rzemińska^3,4^, A. Skoczowski^1,5^, K. Strzałka^3,4^, M. Szaleniec^2^

*^1^Institute of Biology, Pedagogical University of Cracow, Podchorążych 2, 30-084 Krakow, Poland;*

*^2^Jerzy Haber Institute of Catalysis and Surface Chemistry, Polish Academy of Sciences, Niezapominajek 8, 30-239 Krakow, Poland;*

*^3^Faculty of Biochemistry, Biophysics and Biotechnology, Jagiellonian University, Gronostajowa 7, 30-387 Krakow, Poland;*

*^4^Malopolska Centre of Biotechnology, Jagiellonian University, Gronostajowa 7A, 30-387 Krakow, Poland;*

^5^The Franciszek Górski *Institute of Plant Physiology Polish Academy of Sciences, Niezapominajek 21, 30-239 Krakow, Poland.*

Table S1. The experimentally determined reaction enthalpies and log Keq estimated for reactor system according to [1].

| name | ΔH [kJ/mol] | log K_eq_ |
| --- | --- | --- |
| acetophenone | -18.94 | 2.24 |
| acetophenone | -17.58 |  |
| 4'-chloroacetophenone | -18.91 | 3.48 |
| 4'-chloroacetophenone | -20.45 |  |
| 4'-chloroacetophenone | -21.59 |  |
| 4'-hydroxyacetophenone | -9.54 | 1.43 |
| 4'-hydroxyacetophenone | -9.73 | 1.46 |
| 4'-bromoacetophenone | -15.56 | 3.41 |
| 4'-bromoacetophenone | -18.03 |  |
| 4'-bromoacetophenone | -14.44 |  |
| 4'-bromoacetophenone | -17.00 |  |
| 4'-nitroacetophenone | -24.01 |  |
| 4'-nitroacetophenone | -24.86 |  |
| 4-acetylpyridine | -28.75 |  |
| 4-acetylpyridine | -28.91 |  |
| 2-acetylpyridine | -23.13 |  |
| 2-acetylpyridine | -22.00 |  |
| 2,2-di-chloroacetophenone | -36.07 |  |
| 2,2-di-chloroacetophenone | -35.16 |  |
| 2-chloroacetophenone | -29.93 |  |
| 2-chloroacetophenone | -28.38 |  |
| 4'-fluoroacetophenone | n.d | 2.81 |
| 4'-fluoroacetophenone | n.d | 2.15 |
| 4'-ethylacetophenone | n.d | 1.92 |
| 4'-methoxyacetophenone | n.d | 1.75 |
| 4'-methoxyacetophenone | n.d | 1.06 |
| 4'-methoxyacetophenone | n.d | 1.15 |

Table S2. The experimental values of the initial specific activities for all investigated substrates and ln k_cat_ calculated assuming 26.687 kDa mass of the PEDH catalytic subunit.

| **Substrate** | **Specific activity [μmol·min^-1^·mg^-1^]** | **ln k_cat_** |
| --- | --- | --- |
| acetophenone | 1.68 | -0.29 |
| acetophenone | 1.51 | -0.40 |
| acetophenone | 1.41 | -0.47 |
| acetophenone | 1.58 | -0.35 |
| acetophenone | 1.55 | -0.37 |
| acetophenone | 1.65 | -0.31 |
| 4'-chloroacetophenone | 2.31 | 0.03 |
| 4'-chloroacetophenone | 2.60 | 0.15 |
| 4'-chloroacetophenone | 2.40 | 0.07 |
| 4'-chloroacetophenone | 2.37 | 0.05 |
| 4'-hydroxyacetophenone | 0.75 | -1.10 |
| 4'-hydroxyacetophenone | 0.54 | -1.42 |
| 4'-hydroxyacetophenone | 0.72 | -1.14 |
| 4'-hydroxyacetophenone | 0.64 | -1.26 |
| 4'-hydroxyacetophenone | 0.58 | -1.35 |
| 4'-hydroxyacetophenone | 0.58 | -1.36 |
| 4'-hydroxyacetophenone | 0.68 | -1.20 |
| 4'-bromoacetophenone | 3.26 | 0.37 |
| 4'-bromoacetophenone | 3.09 | 0.32 |
| 4'-bromoacetophenone | 2.74 | 0.20 |
| 4'-bromoacetophenone | 3.02 | 0.29 |
| 4'-bromoacetophenone | 2.64 | 0.16 |
| 4'-nitroacetophenone | 9.44 | 1.43 |
| 4'-nitroacetophenone | 7.55 | 1.21 |
| 4'-nitroacetophenone | 8.31 | 1.31 |
| 4-acetylpyridine | 13.68 | 1.81 |
| 4-acetylpyridine | 14.49 | 1.86 |
| 4-acetylpyridine | 12.52 | 1.72 |
| 4-acetylpyridine | 16.38 | 1.99 |
| 4-acetylpyridine | 14.87 | 1.89 |
| 4-acetylpyridine | 16.86 | 2.01 |
| 2-acetylpyridine | 9.99 | 1.49 |
| 2-acetylpyridine | 12.05 | 1.68 |
| 2-acetylpyridine | 13.38 | 1.78 |
| 2-acetylpyridine | 13.98 | 1.83 |
| 2’,2’-di-chloroacetophenone | 9.52 | 1.44 |
| 2’,2’-di-chloroacetophenone | 9.77 | 1.47 |
| 2’,2’-di-chloroacetophenone | 10.67 | 1.56 |
| 2’,2’-di-chloroacetophenone | 10.50 | 1.54 |
| 2’,2’-di-chloroacetophenone | 10.46 | 1.54 |
| 2’-chloroacetophenone | 3.11 | 0.32 |
| 2’-chloroacetophenone | 2.97 | 0.28 |
| 2’-chloroacetophenone | 3.45 | 0.43 |
| 2’-chloroacetophenone | 2.92 | 0.26 |
| 2’-chloroacetophenone | 3.33 | 0.39 |

Figure S3. Experimentally determined activation energies

|  |  | Activation energy [kJ/mol] |
| --- | --- | --- |
| 4’-H |  | 73.29 ±4.33 |
| 2,2-di-Cl |  | 63.83 ± 6.89 |
| 2-etpy |  | 44.98 ±1.9 |


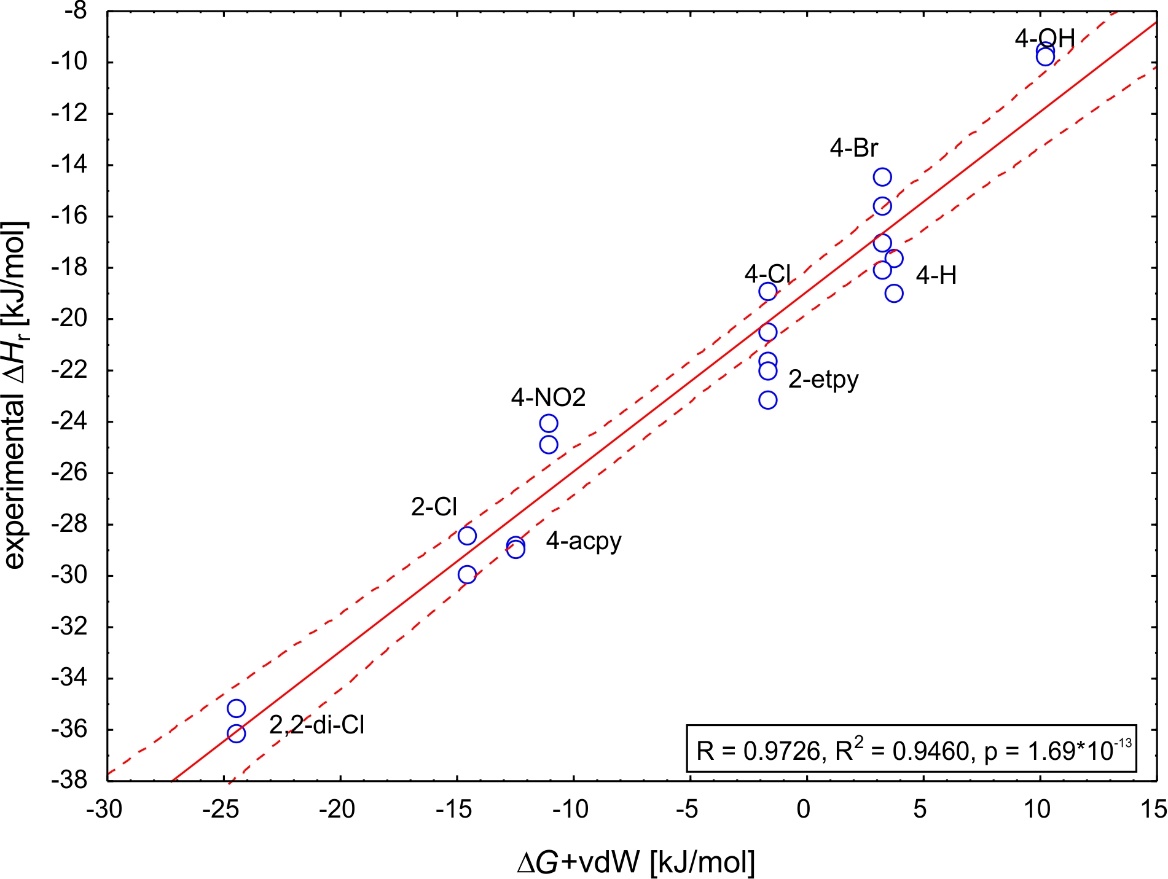


**Figure S1**. Correlation plot of the experimental reaction enthalpy ΔH_r_ with ΔG+vdW (R^2^ =0.9460). The solid line represent linear fit while dashed line represents 95% confidence range.


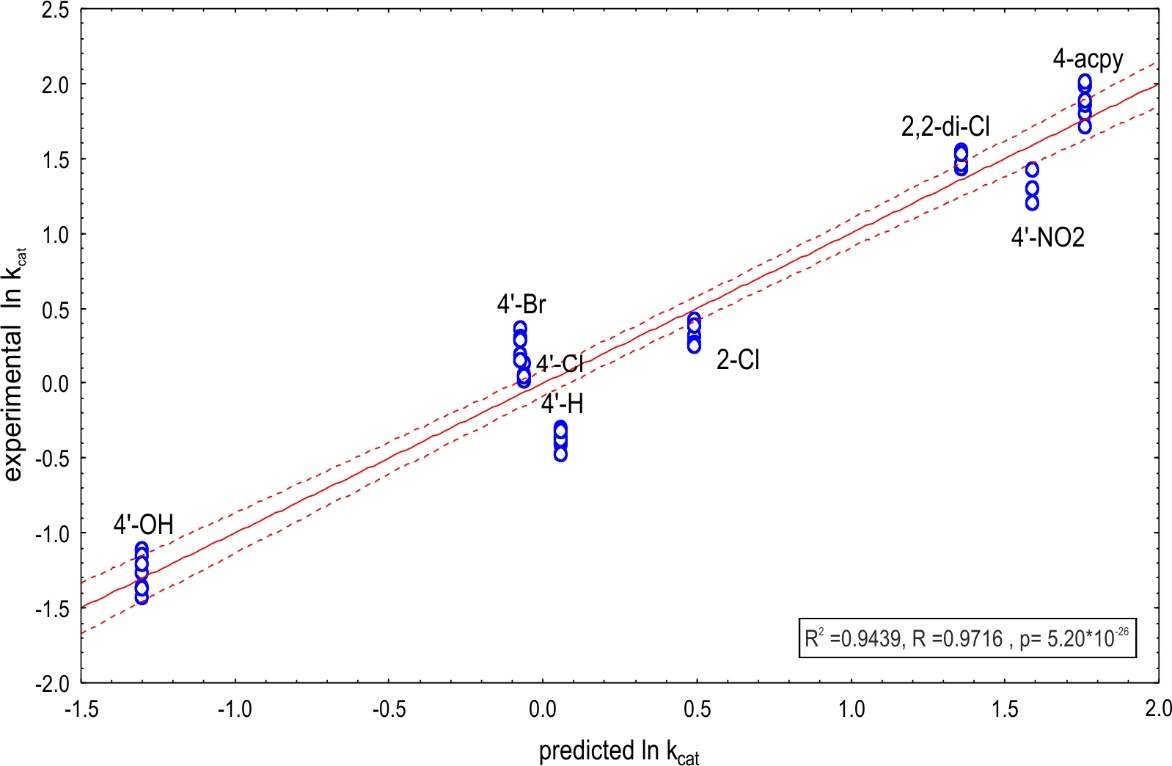


**Figure S2**. Correlation plot of the experimental ln k_cat_ with predicted ln kcat by MLR1 (R^2^ =0.9439). The solid line represent linear fit while dashed line represents 95% confidence range.

**MLR2 model that predicts ln kcat for the whole dataset including 2-acpy**

ln k_cat_ = -0.0815 (±0.009) ΔG^#^ + -7.83 (±1.45) d(ryb-O—H-NH_2_-Lys) -11.784 (±3.12) d(H—C) +37.16 (±5.51)

n=45, R^2^= 0.8753, corr. R^2^= 0.8661, F=95.936, p=1.42*10^-18^, estimation error=0.397


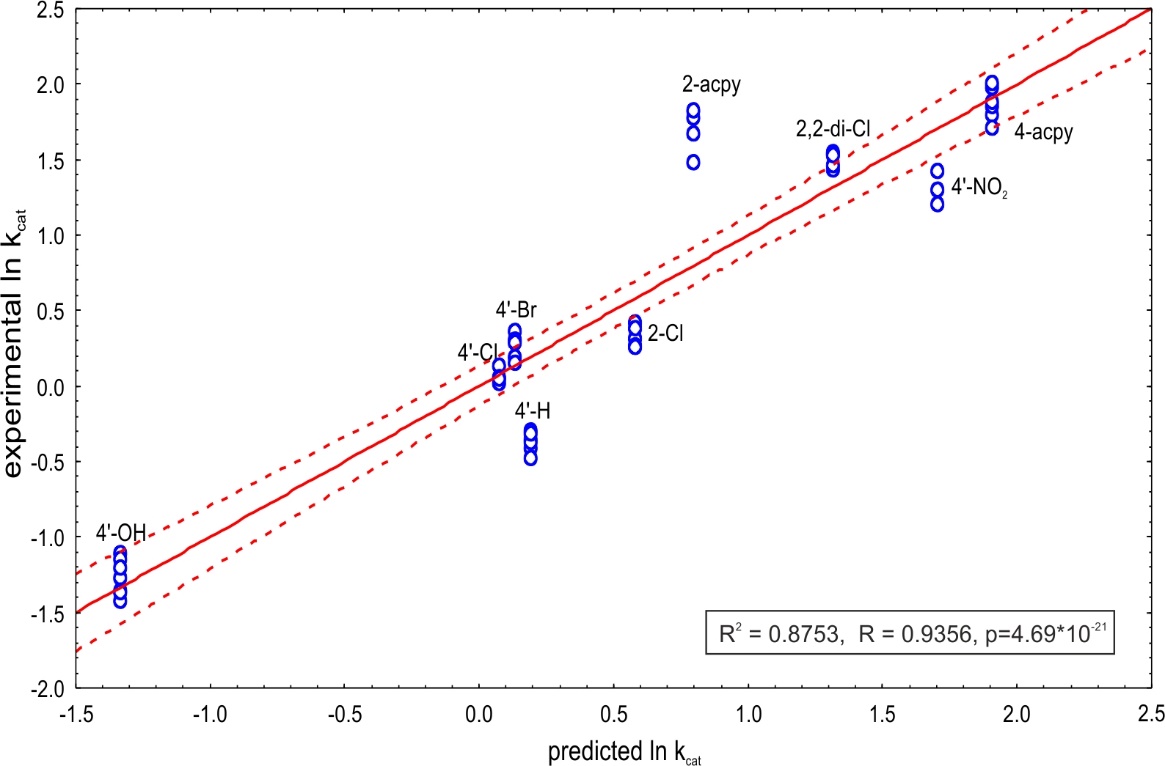


**Figure S3**. Correlation plot of the experimental ln k_cat_ with predicted ln kcat by MLR2 (R^2^=0.8753). The solid line represent linear fit while dashed line represents 95% confidence range.

| **Model** | **ES** | **TS** | **EP** |
| --- | --- | --- | --- |
| 4’-H high pH X3LYP | 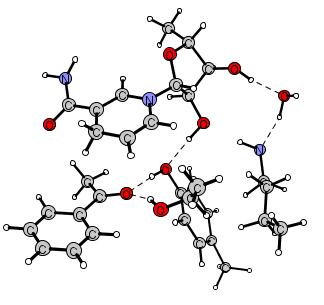 | 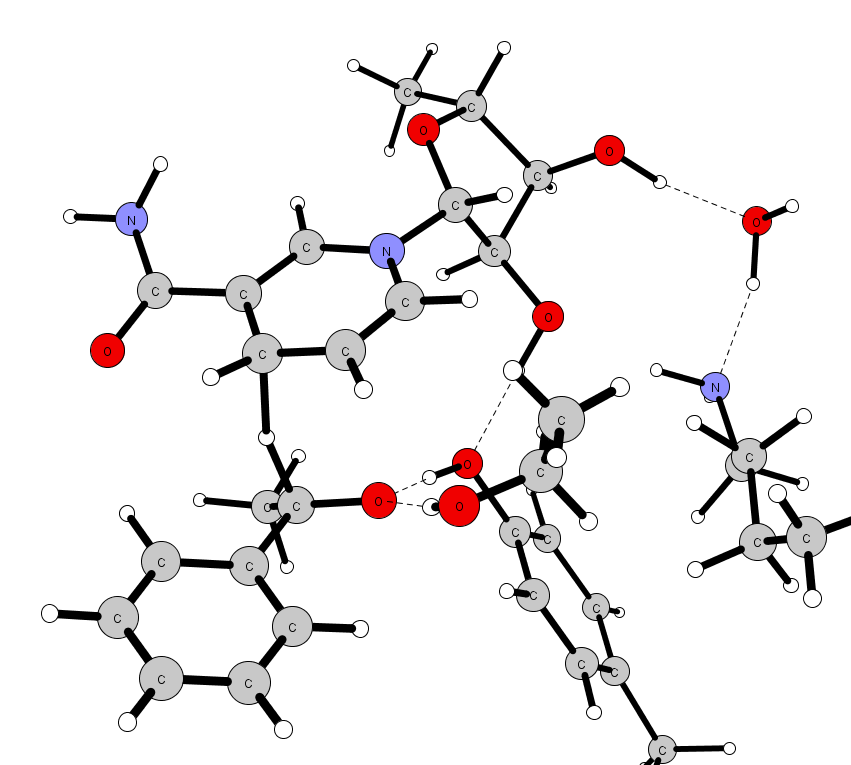 | 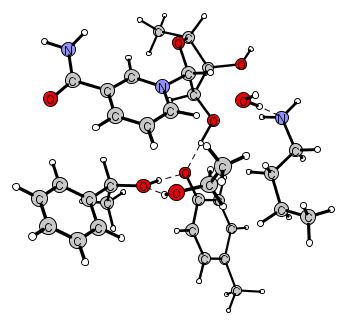 |
| 4’-H low pH  X3LYP | 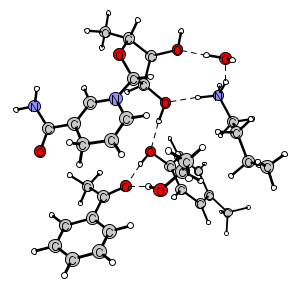 | 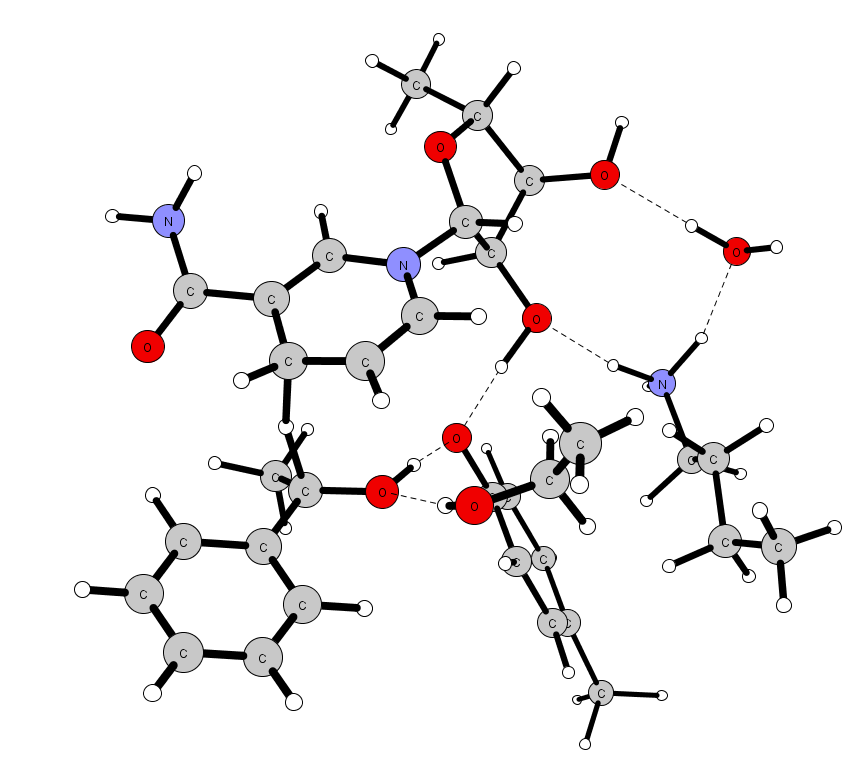 | 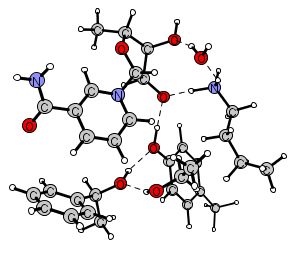 |
| 4’-H high pH | 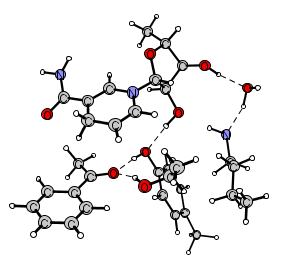 | 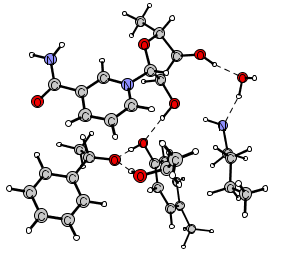 | 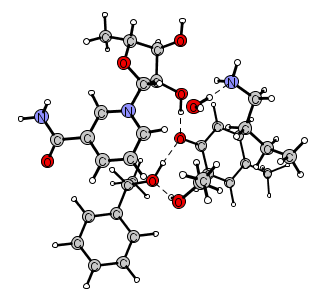 |
| 4’-H low pH | 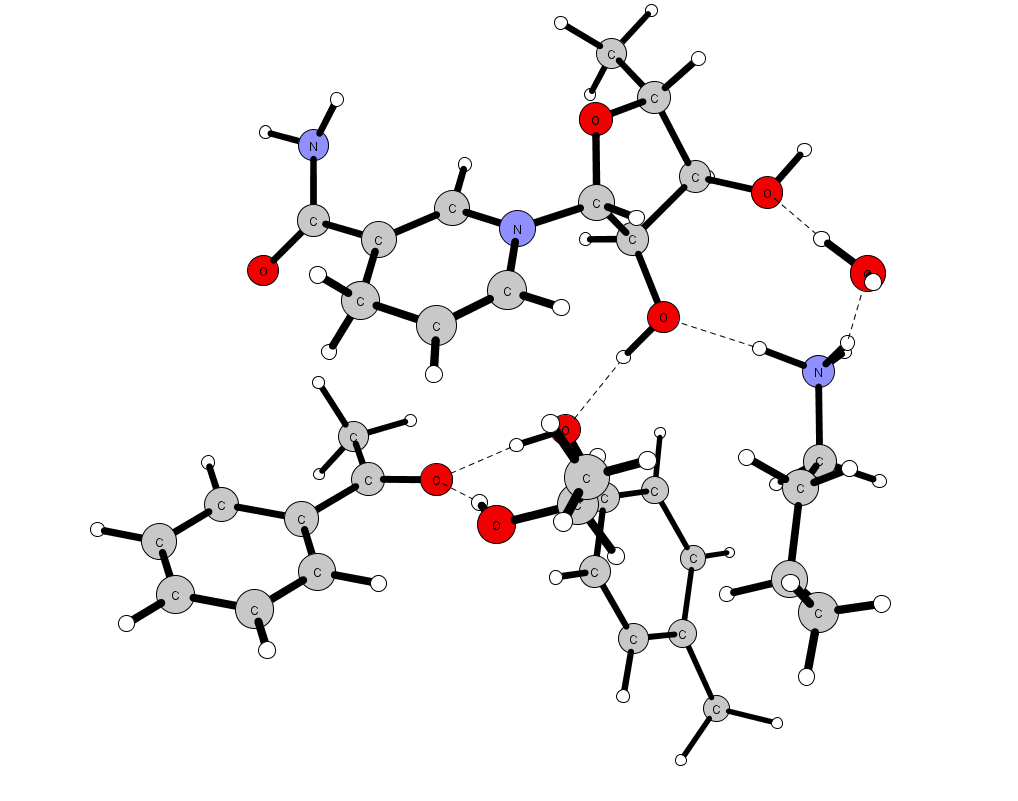 | 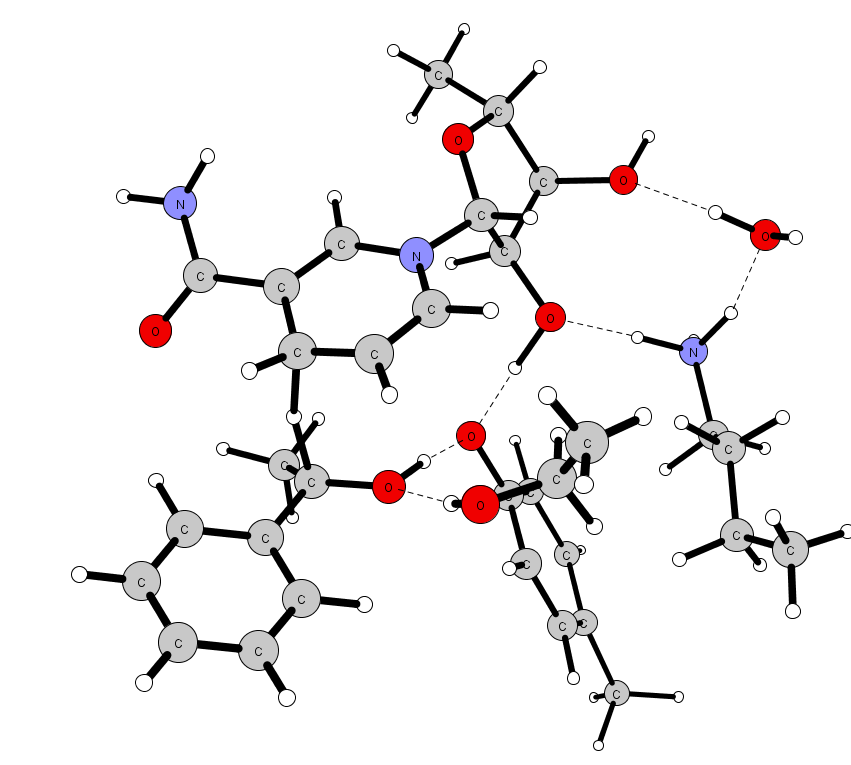 | 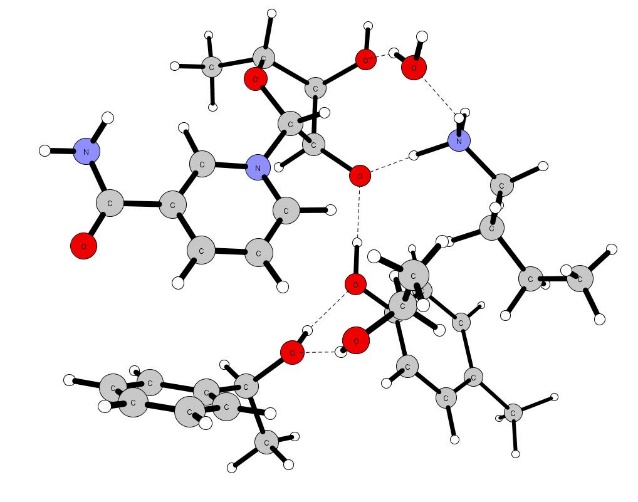 |
| 4’-OH | 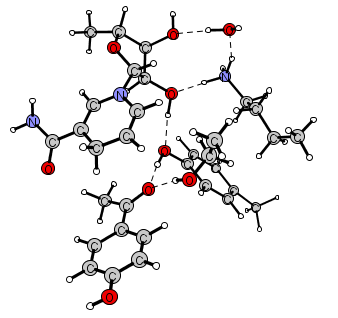 | 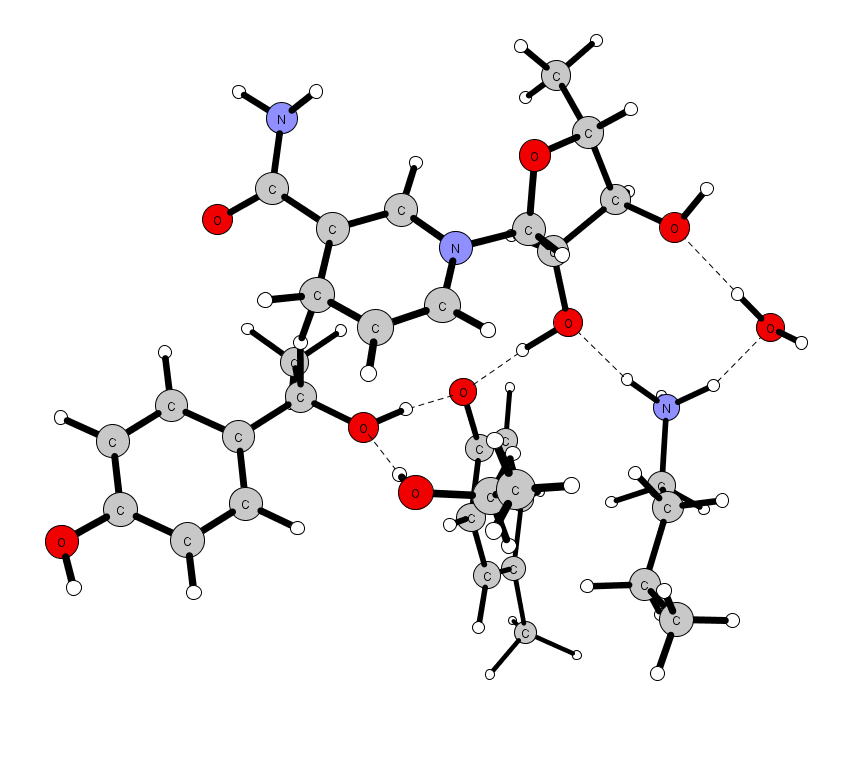 | 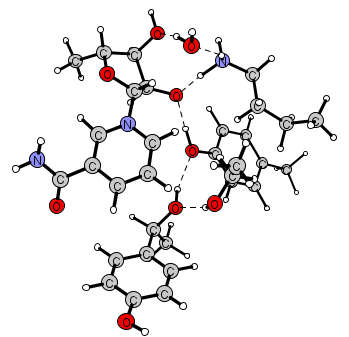 |
| 4’-Cl | 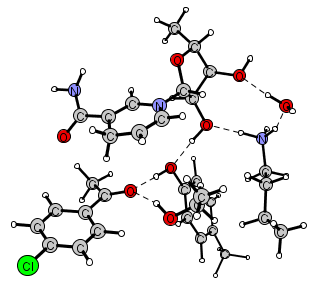 | 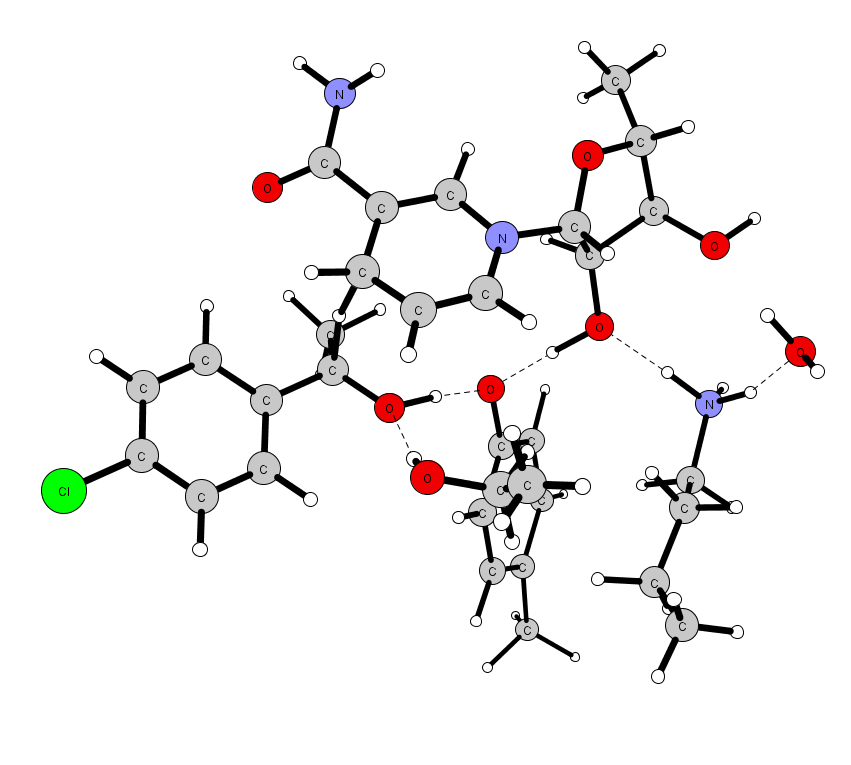 | 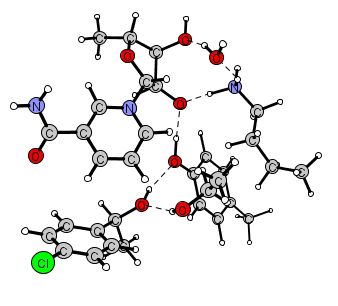 |
| 4’-Br | 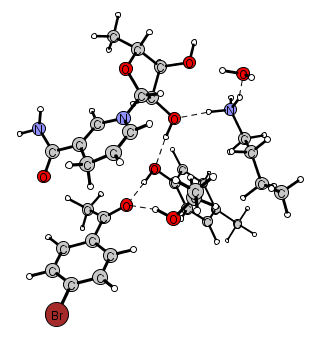 | 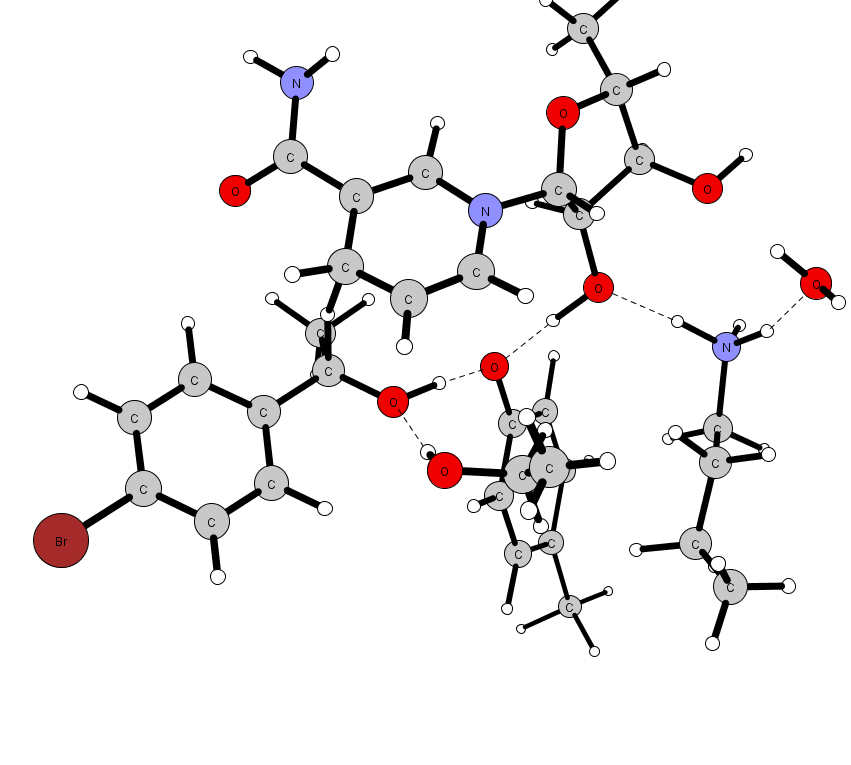 | 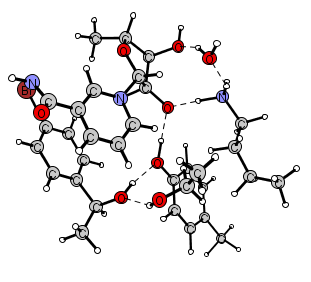 |
| 4’-NO_2_ | 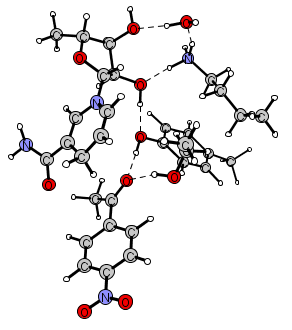 | 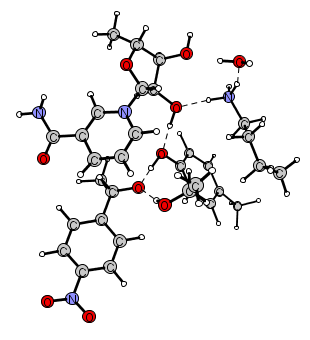 | 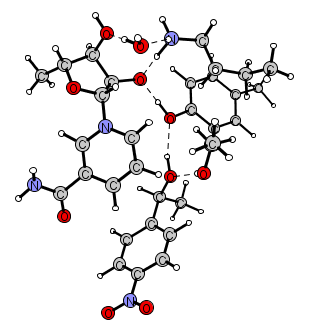 |
| 4-acpy | 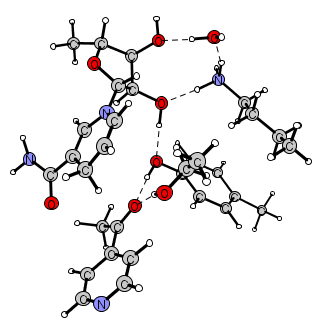 | 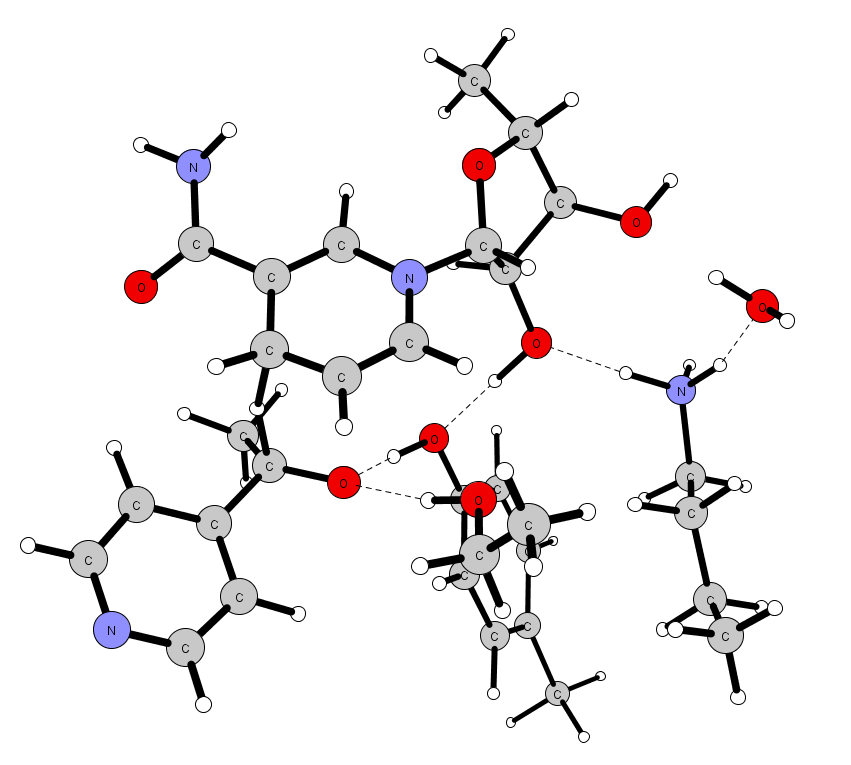 | 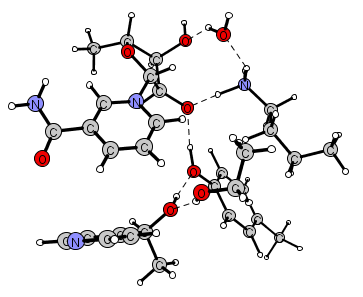 |
| 2-acpy syn | 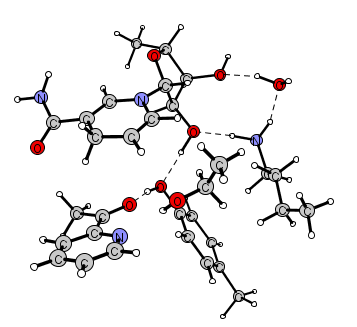 | 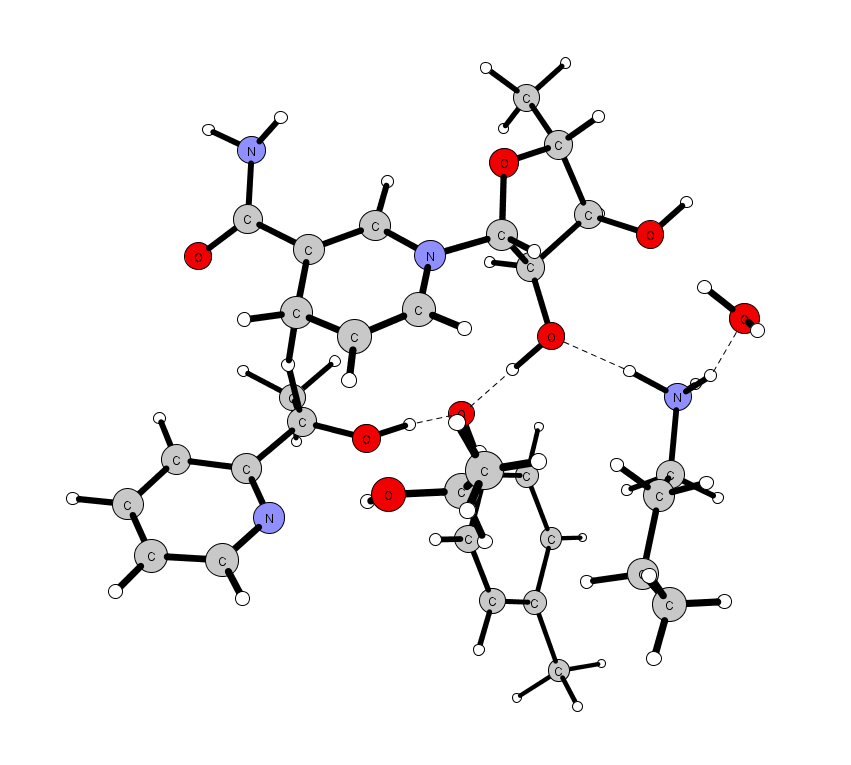 | 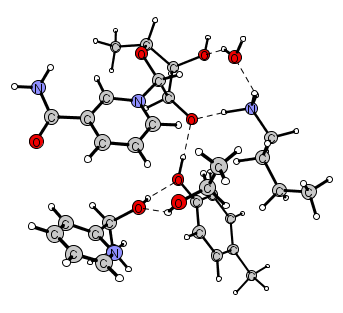 |
| 2-acpy anti | 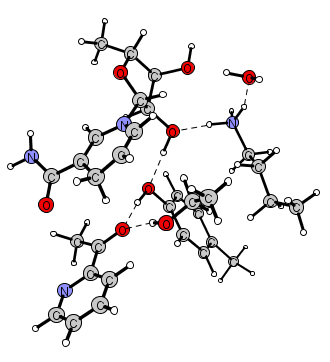 | 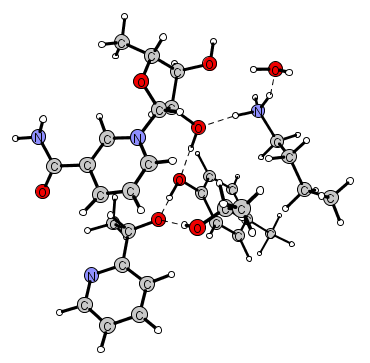 | 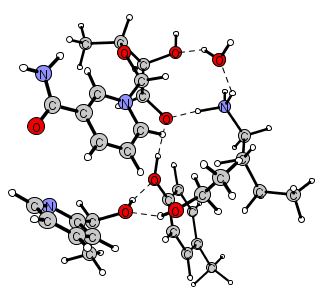 |
| 2-Cl | 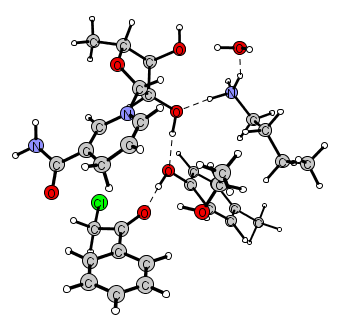 | 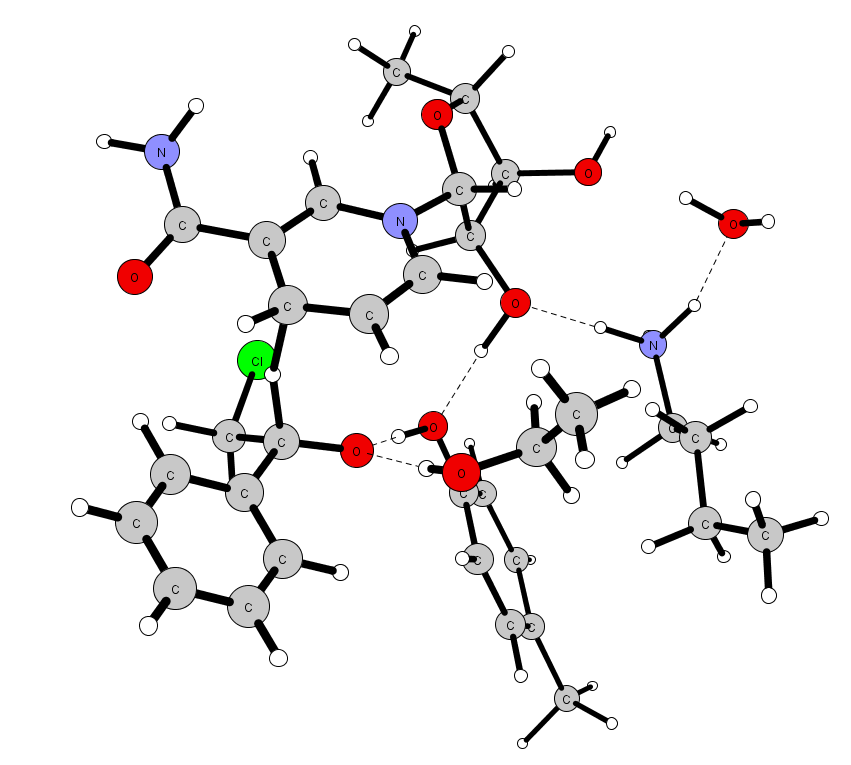 | 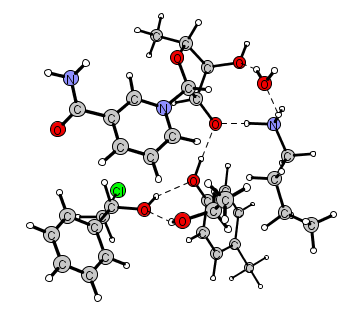 |
| 2,2-di-Cl | 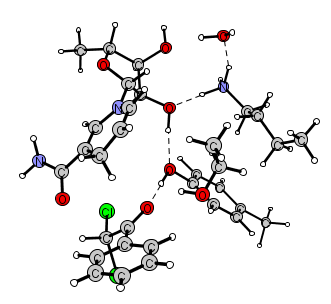 | 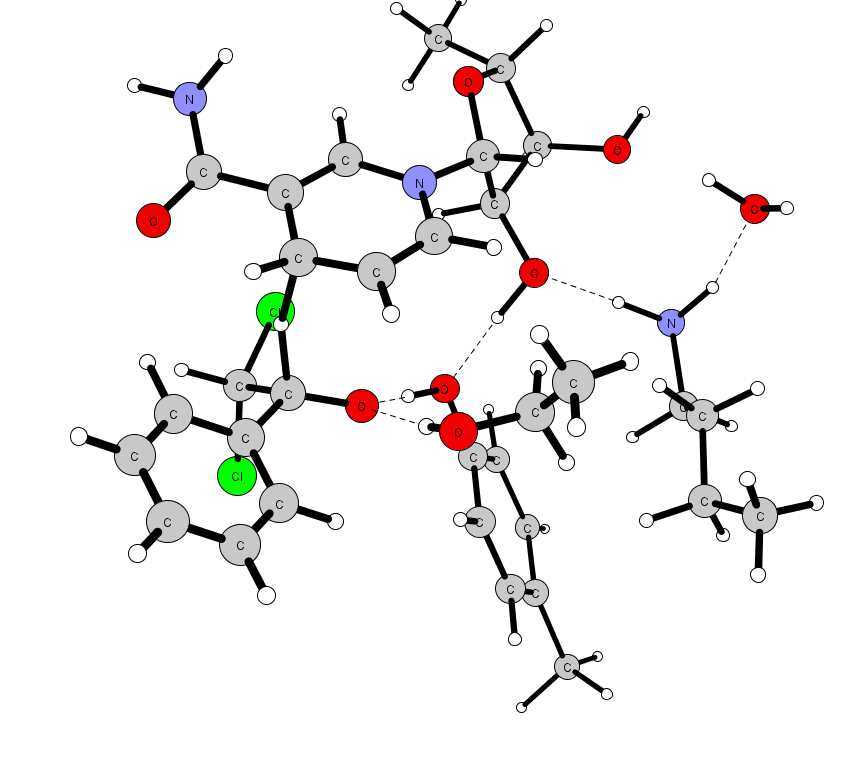 | 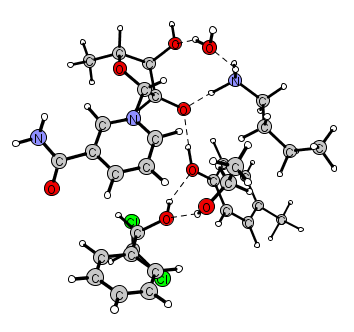 |
| 4’-MeO | 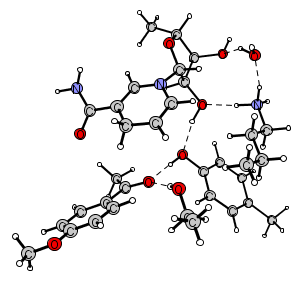 | 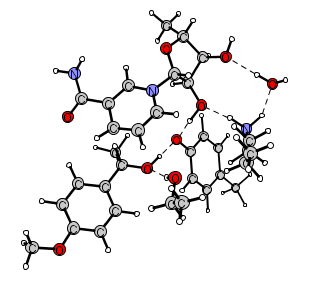 | 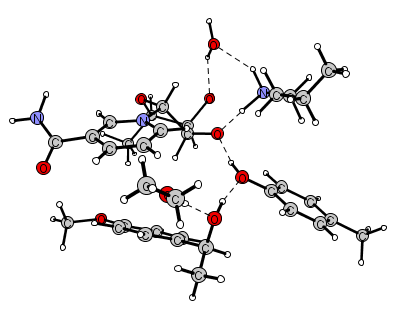 |
| 4’-F | 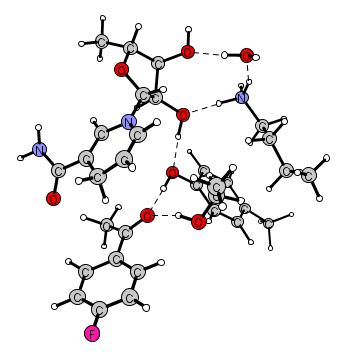 | 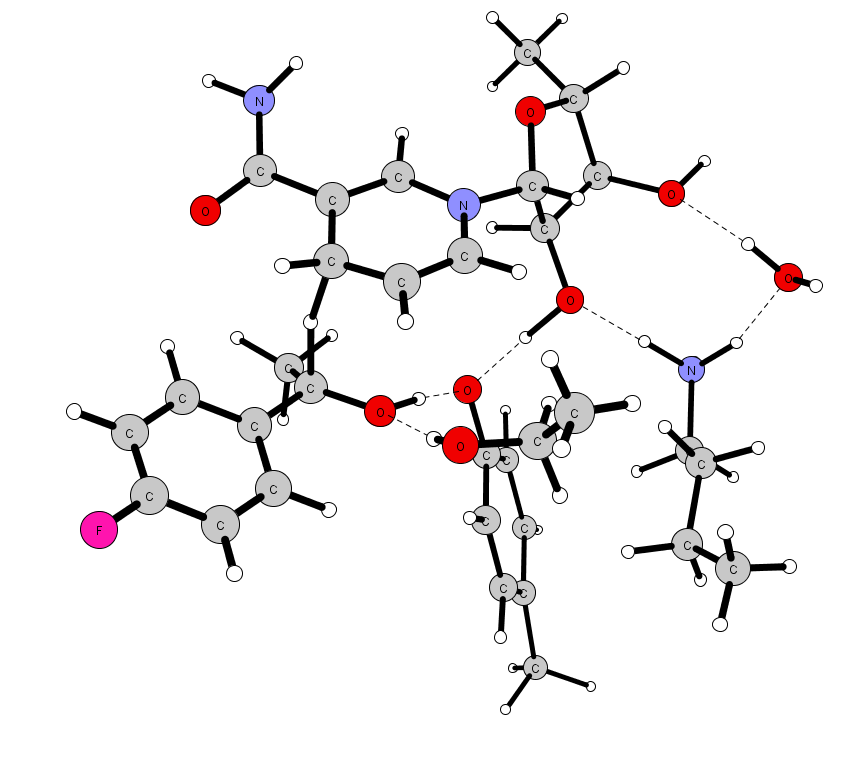 | 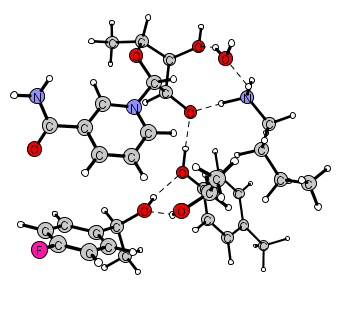 |
| 4’-Et | 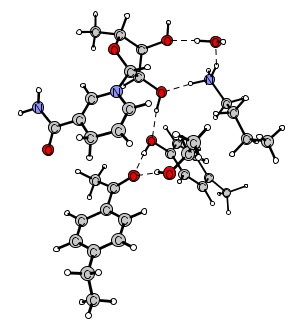 | 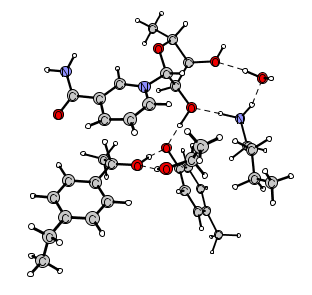 | 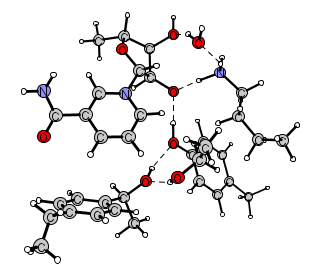 |

References

1. Dudzik A, Snoch W, Borowiecki P, Opalinska-Piskorz J, Witko M, Heider J, Szaleniec M (2015) Appl Microbiol Biotechnol 99(12):5055-5069
